# MiR-494 induces metabolic changes through G6pc targeting and modulates sorafenib response in hepatocellular carcinoma

**DOI:** 10.1186/s13046-023-02718-w

**Published:** 2023-06-10

**Authors:** Christian Bergamini, Ilaria Leoni, Nicola Rizzardi, Mattia Melli, Giuseppe Galvani, Camelia Alexandra Coada, Catia Giovannini, Elisa Monti, Irene Liparulo, Francesca Valenti, Manuela Ferracin, Matteo Ravaioli, Matteo Cescon, Francesco Vasuri, Fabio Piscaglia, Massimo Negrini, Claudio Stefanelli, Romana Fato, Laura Gramantieri, Francesca Fornari

**Affiliations:** 1 Department of Pharmacy and Biotechnology, University of Bologna, 40126 Bologna, Italy; 2 Centre for Applied Biomedical Research - CRBA, University of Bologna, Policlinico di Sant’Orsola, 40138 Bologna, Italy; 3 Department for Life Quality Studies, University of Bologna, 47921 Rimini, Italy; 4 Department of Medical and Surgical Sciences, University of Bologna, 40138 Bologna, Italy; 5 IRCCS Azienda Ospedaliero-Universitaria di Bologna, 40138 Bologna, Italy; 6 Hepato-biliary Surgery and Transplant Unit, IRCCS Azienda Ospedaliero-Universitaria di Bologna, 40138 Bologna, Italy; 7 Department of Pathology, IRCCS Azienda Ospedaliero-Universitaria di Bologna, 40138 Bologna, Italy; 8 Division of Internal Medicine, Hepatobiliary and Immunoallergic Diseases, IRCCS Azienda Ospedaliero-Universitaria di Bologna, Via Massarenti, 9, 40138 Bologna, Italy; 9 Department of Morphology, Surgery and Experimental Medicine, University of Ferrara, 44100 Ferrara, Italy

**Keywords:** HCC, microRNA, miR-494, G6pc, Metabolism, Biomarker, Sorafenib

## Abstract

**Background:**

Metabolic reprogramming is a well-known marker of cancer, and it represents an early event during hepatocellular carcinoma (HCC) development. The recent approval of several molecular targeted agents has revolutionized the management of advanced HCC patients. Nevertheless, the lack of circulating biomarkers still affects patient stratification to tailored treatments. In this context, there is an urgent need for biomarkers to aid treatment choice and for novel and more effective therapeutic combinations to avoid the development of drug-resistant phenotypes. This study aims to prove the involvement of miR-494 in metabolic reprogramming of HCC, to identify novel miRNA-based therapeutic combinations and to evaluate miR-494 potential as a circulating biomarker.

**Methods:**

Bioinformatics analysis identified miR-494 metabolic targets. QPCR analysis of glucose 6-phosphatase catalytic subunit (G6pc) was performed in HCC patients and preclinical models. Functional analysis and metabolic assays assessed G6pc targeting and miR-494 involvement in metabolic changes, mitochondrial dysfunction, and ROS production in HCC cells. Live-imaging analysis evaluated the effects of miR-494/G6pc axis in cell growth of HCC cells under stressful conditions. Circulating miR-494 levels were assayed in sorafenib-treated HCC patients and DEN-HCC rats.

**Results:**

MiR-494 induced the metabolic shift of HCC cells toward a glycolytic phenotype through G6pc targeting and HIF-1A pathway activation. MiR-494/G6pc axis played an active role in metabolic plasticity of cancer cells, leading to glycogen and lipid droplets accumulation that favored cell survival under harsh environmental conditions. High miR-494 serum levels associated with sorafenib resistance in preclinical models and in a preliminary cohort of HCC patients. An enhanced anticancer effect was observed for treatment combinations between antagomiR-494 and sorafenib or 2-deoxy-glucose in HCC cells.

**Conclusions:**

MiR-494/G6pc axis is critical for the metabolic rewiring of cancer cells and associates with poor prognosis. MiR-494 deserves attention as a candidate biomarker of likelihood of response to sorafenib to be tested in future validation studies. MiR-494 represents a promising therapeutic target for combination strategies with sorafenib or metabolic interference molecules for the treatment of HCC patients who are ineligible for immunotherapy.

**Supplementary Information:**

The online version contains supplementary material available at 10.1186/s13046-023-02718-w.

## Background

Hepatocellular carcinoma (HCC) ranks fourth among the most lethal cancers [1], with metabolic syndrome gaining more relevance among HCC risk factors [2]. Dysmetabolic liver diseases include nonalcoholic fatty liver disease (NAFLD) and nonalcoholic steatohepatitis (NASH) which can progress to cirrhosis in 10–20% of cases, and to HCC without overt cirrhosis [3]. Reverting dysmetabolism reduces HCC risk [4, 5]. Metabolic changes in neoplastic hepatocytes give them a survival and growth advantage in challenging conditions, altering tumor microenvironment, and impairing immune surveillance [6]. Tumor cells are metabolically reprogrammed to fuel cell proliferation and to produce energetic and metabolic precursors, mostly by increasing glucose uptake and flux through aerobic glycolysis and anabolic pathways. Metabolic reprogramming is a well-known hallmark of cancer [7] and a very early event in preclinical models of hepatocarcinogenesis [8] and is involved in drug resistance of HCC cells [9].

The liver plays a central role in glucose homeostasis controlling its storage, synthesis, and bloodstream concentration. Glucose-6 phosphate (G6P) is the first intermediate of glucose metabolism linking crucial metabolic pathways such as glycolysis, glycogenesis, de novo lipogenesis, and pentose phosphate pathway (PPP) [10]. Glucose-6 phosphatase (G6Pase) is a multi-subunit complex located in the endoplasmic reticulum membrane catalyzing the de-phosphorylation of G6P to free glucose for energy supply and glycaemia regulation [11]. Genetic mutations of the G6Pase catalytic subunit (*G6PC*) have been identified in a rare genetic disorder namely glycogen storage disease type Ia (GSD-Ia). In these patients, G6P accumulation is redirected to downstream metabolic pathways leading to aberrant lactate production and ectopic lipid accumulation, elevating the risk for liver cancer development [12]. HCC derived from liver KO G6pc^−/−^ mice showed increased glycolysis and decreased oxidative phosphorylation (OXPHOS) accounting for metabolic shift or ‘Warburg effect’ [13], which is characterized by high rates of glycolysis and lactic acid fermentation that occur regardless of oxygen levels.

Among molecular mechanisms contributing to metabolic alterations of cancer cells, HCC-specific microRNAs (miRNAs) were shown to modulate critical metabolic genes [14]. Of note, the liver-specific miR-122 regulates cholesterol and lipid biosynthesis [15, 16]. Similarly, miR-221 overexpression in the liver of transgenic mice leads to lipid accumulation and variable extents of steatohepatitis [17]. Recent studies highlighted the involvement of oncogenic/tumor suppressor miRNAs in liver tumorigenesis through the regulation of metabolic targets [18, 19]. We reported the upregulation of miR-494 in a subgroup of HCCs with stem cell properties and showed its effects in sorafenib resistance through the activation of mammalian target of rapamycin (mTOR) pathway [20].

Here, we investigated the role of miR-494 in metabolic rewiring of HCC cells and identified G6pc as its target gene contributing to confer metabolic plasticity to cancer cells. In two HCC patient cohorts, lower G6pc levels associated with aggressive tumor features and decreased overall survival. Finally, we identified miR-494 as a circulating biomarker associated with sorafenib resistance in advanced HCC patients and reported the therapeutic potential of antimiR-494 oligonucleotides in combination with sorafenib and metabolic drugs.

## Methods

### HCC patient cohorts

HCC and surrounding liver tissues were obtained from patients undergoing liver surgery for HCC at the Department of Surgery and Transplantation, IRCCS Azienda Ospedaliero-Universitaria of Bologna (local ethics committee approval: 138/2015/O/Tess). Tissue samples were collected at surgery and stored at -80 °C. The clinical characteristics of patients are shown in Supplementary Table 1. Circulating miR-494 levels were tested in the sorafenib-treated HCC patient cohort (local ethics committee approval: 271/2012/O/Oss). The clinical characteristics of advanced patients are detailed in Supplementary Table 2. Blood samples were collected before treatment and processed as previously described [21]. Informed written consent was obtained from all patients enrolled in the study. Data from The Cancer Genome Atlas Liver Hepatocellular Carcinoma project (TCGA-LIHC – hereinafter referred to as TCGA-HCC) cohort include 374 HCC and 50 non-tumor liver samples. Differential expression analysis was performed in R software v. 4.2.1. [22] using the DeSeq2 package [23]. TCGA HCC patients were ordered based on the level of miR-494 and then divided into high and low miR-494 expressing tumors. Venn diagrams were generated using the VennDiagram R package. Identification of miR-494 targets was done using TargetScan release 7. Identification of mitochondrial genes was done using the MitoCarta database as a reference [24].

### HCC animal models

Diethylnitrosamine (DEN)-induced HCC rats (*N* = 18) and xenograft mice (*N* = 13) were established and treated as previously described [20, 25]. Treated rats (*N* = 12) received sorafenib (10 mg/kg) for 21 days by intra-gastrical administration starting when the major diameter of HCC nodules was about of 3–5 mm long as evaluated by ultrasound monitoring. The animals were euthanized the day after the end of treatment. The protocols were approved by local ethics committee of Bologna University (xenograft mice, protocol N. 23–79-2014) and by Italian Minister of Health (DEN-HCC rats, protocol N.421/2016-PR).

### Microarray analysis

RNAs from the DEN-HCC rat model (11 HCCs and 9 matched non-tumor livers) and 2 normal livers from healthy rats used as controls were hybridized on Agilent rat gene expression microarrays (SurePrint G3 Rat GE 8 × 60 K Microarray #G4853A, Agilent Technologies). One-color gene expression was performed according to the manufacturer’s procedure as previously described [26]. Raw data are available in ArrayExpress repository database (accession number E-MTAB-11977) at EMBL-EBI (www.ebi.ac.uk/arrayexpress). Raw data were analyzed with GeneSpring software v.14.9 (Agilent Technologies).

### HCC cell lines and treatments

HCC-derived cell lines were cultured and stably infected with retroviral vectors as previously described [20, 27]. Specifically, the DNA sequence of miR-494 precursor was inserted within XhoI cloning sites of pMXs-miR-GFP/Puro retroviral expression vector according to manufacturer’s instructions (Cell Biolabs) to obtain miR-494 overexpressing vector (pMXs-miR-494). The control vector (pMXs) contains a scramble shRNA sequence. Retroviral particles, obtained by transfecting these vectors into Phoenix A packaging cells, were used to infect Huh-7 cells that were selected by adding puromycin (10 µg/ml) 48 h after infection. HCC cell lines were transiently transfected with 80 nM of pre-miR-494 or antimiR-494 or Negative Control precursor and inhibitor miRNAs (Ambion) for 48 h. HIF-1A silencing was obtained by using Dicer-substrate short interfering RNAs (DsiRNAs, IDT) that are 27mer duplex RNAs that demonstrated increased potency in RNA interference (Supplementary Table 3). Cells were treated with 5.0 µM of sorafenib (Bayer) for 48 or 72 h. Metabolic inhibitors were used at the following concentrations: 2-deoxy-glucose (2-DG) 10 mM; antimycin A (AA) 20 µM (Sigma-Aldrich). For starvation conditions, growth medium was deprived of FBS. No glucose medium was added with 10% dialyzed FBS and pyruvate 1 mM. G6PC (Myc-DDK-tagged) overexpression vector (ID: RC215623) and control vector pCMV6 were from Origene. Incucyte Live-Cell Analysis System (Sartorius) was used for real time monitoring of cell growth. Specifically, the phase area per well (µm^2^/well) was quantified at each time point and normalized to T0. Mean ± SD values were used for the graphical representation of growth curves. Experiments were performed in quadruplicate. The formula for doubling time calculation in the 24 h was 24*Ln(2)/Ln(T2/T1), where T1 and T2 represent the cell growth values corresponding to 12 and 36 h, respectively.

### PAS staining

Four-micrometer thick FFPE sections were processed for Periodic Acid Schiff (PAS) staining. Briefly, oxidation was obtained by incubating slides in 0.5% Periodic Acid solution for 5 min whereas staining was performed by adding Schiff’s reagent for 7 min. Counterstaining was obtained with Meyer’s hematoxylin. The glycogen accumulation in HCC cells was analyzed by PAS method with minor modifications [28]. Cells were seeded onto a coverslip in a 6-well plate at 0.1 × 10^5^ cells/well. Subsequently, culture medium was removed and replaced with complete medium or no glucose medium for 24 h. After washing with PBS, cells were fixed in 4% paraformaldehyde at RT for 1 h and washed using distilled water. Samples were incubated for 10 min at RT with Periodic Acid solution, treated with Shiff’s reagent for 20 min at RT in the dark and counterstained with hematoxylin. The coverslips were washed with tap water for 5 min, let dry for 10 min, and then fixed in glycerol based mounting medium on a microscope slide. Images were acquired using a DM750 microscope with ICC50 digital camera (Leica). At least 5 randomly chosen fields (20X magnification) were acquired. To evaluate the PAS signals from images, we subjected the background-corrected images to the commonly used color deconvolution with defined color vectors, and then percentage of cell area staining positive for PAS was determined using ImageJ Software tools (ImageJ, National Institutes of Health).

### Real time PCR

TaqMan MicroRNA Assays (Applied Biosystems) were used to evaluate miRNA expression. RNU6B was used as housekeeping gene for intracellular miRNAs, whereas cel-miR-39 was used for circulating miRNAs [21]. SYBR-green qPCR was used for gene expression analysis, using β-Actin and GAPDH as housekeeping genes (Supplementary Table 3). QPCR experiments were run in triplicate.

### Western blot

Western blot (WB) was used to analyze protein expression by using antibodies reported in Supplementary Table 4. ChemiDoc XRS + (Image Lab Software, Bio-Rad) was employed to acquire and quantify digital images. Two independent experiments were performed.

### Luciferase reporter assay

G6pc 3’-untraslated region was amplified by PCR using primers reported in Supplementary Table 5. Mutagenesis of miR-494 site was performed using QuikChange II Site-Directed Mutagenesis Kit (Agilent Technologies) following the manufacturer's instruction; Sanger sequencing verified the mutated sequence. Dual-Glo luciferase assay system (Promega) was performed in HCC cell lines as previously described [20].

### Spheroids formation and staining assay

Spheroids formation was performed following Wang et al. [29]. In brief, cells were seeded in 35 mm diameter dishes high Bioinert (Ibidi, Germany) at 0.1 × 10^6^ cells/dish density in complete medium supplemented with 25% of Methocel (Sigma-Aldrich) stock solution (0.5 g in 500 ml of complete medium). Spheroids size (µm) was determined by measuring feret’s diameter using standard ImageJ software tools (ImageJ, National Institutes of Health). Intracellular oxygen level was measured using BTP (bis(2-(20-benzothienyl)-pyridinato-N,C30) iridium (acetylacetonate) (Sigma-Aldrich), a fluorescent dye quenched by molecular oxygen [30]. Spheroids were stained with 5 µM BTP in complete medium for 4 h. After this time, spheroids were carefully washed with phenol-red free medium. Images of at least ten spheroids for condition were acquired using a Nikon C1si confocal microscope (Nikon). Fluorescence intensity was analyzed using standard ImageJ software tools.

### Oxygen consumption determination

The oxygen consumption in control (pMXs) and miR-494-overexpressing Huh-7 cells was measured using a thermostatically controlled oxygraph chamber at 37 °C equipped with Clark electrode (Instech Mod. 203, USA) as previously described [31]. Briefly, 0.8 × 10^6^ cells were seeded in a Petri dish and grown for 48 h in standard culture conditions. Then, cells were washed in NaCl 0,9%, trypsinized, and resuspended in complete medium (RPMI). Endogenous respiration was measured in RPMI (basal respiration), after the addition of 1 µM of the ATPase inhibitor oligomycin A (non-phosphorylating respiration), and of 500 nM of the uncoupler carbonyl cyanide 4-(trifluoromethoxy) phenylhydrazone (FCCP) (maximal respiration). The respiratory control ratio (RCR) was obtained by dividing the maximum oxygen consumption rate (FCCP) by respiration rate after oligomycin A addition. All data were expressed as nmoles O_2_·min^−1^·mg proteins^−1^.

### Mitochondrial transmembrane potential (ΔΨm) and network morphology analysis

ΔΨm was assessed using the mitochondrial fluorescent probes Tetramethylrhodamine methyl ester (TMRM λex558 nm; λem578 nm; Thermo Fisher). Mitochondrial network morphology was visualized using the fluorescent probe Mitotracker Green (MTG, λexc490 nm, λem516 nm; Thermo Fisher). Cells were seeded in µ-8 wells (Ibidi) and were stained with 50 nM TMRM and 100 nM MTG for 30 min in complete medium [32]. After this time, the staining medium was removed, and cells were washed with Hank’s balanced salt solution (HBSS). Images were acquired using a Nikon C1si confocal microscope (Nikon). TMRM fluorescence intensity was measured using ImageJ software. Mitochondrial network morphology was calculated using MiNA ImageJ plugin [33]. At least 5 randomly chosen fields were acquired and analyzed for each condition.

### Succinate dehydrogenase and citrate synthase activity

The succinate dehydrogenase (Complex II, SDH) and the citrate synthase (CS) activities were measured as described in Spinazzi et al. [34] using a UV–vis spectrophotometer (V-750, JASCO) equipped with a cuvette stirring device and thermostatic control. For Complex II activity determination, cells were collected by centrifugation, washed with PBS, and suspended in 20 mM hypotonic potassium phosphate buffer (pH 7.5) followed by three cycles of freeze-thawing. Complex II activity was measured by following the reduction of 80 μM 2,6- Dichlorophenolindophenol sodium salt hydrate (DCPIP; Sigma) at λ = 600 nm (ε = 19.1 mmol ^−1^ cm ^– 1^) in the presence of 25 mM potassium phosphate buffer, 300 μM KCN, 1 mg ml^−1^ fatty acid–free bovine serum albumine (BSA), 20 mM succinate, 150 µg of cell lysate and 50 μM decylbenzoquinone (DB). The specific Complex II activity was obtained by subtracting the carboxine-insensitive DCPIP reduction rate. CS activity was followed at λ = 412 nm in 100 mM TRIS buffer (pH 8), 0.1% Triton, 0.1 mM acetyl-coA, 0.5 mM oxalacetate and 0.1 mM 5,5’-dithiobis-2-nitrobenzoic acid (DTNB, ε = 13.6 mmol ^−1^ cm ^−1^) and 30 µg of cell lysate at 30 °C. Data were normalized to protein content determined by the Lowry method.

### Extracellular lactate determination

The lactate amount was assessed by HPLC following Liparulo et al. [31] with minor modifications. Control (pMXs) and miR-494-overexpressing Huh-7 cells were cultured in a six well plate until 80% confluence. The culture medium was collected and diluted 1:5 in the mobile phase consisting of 50 mM KH2PO4, pH 2.4 and centrifuged at 14,000 g for 5 min at 4 °C. The supernatant was collected and injected in an HPLC system (Agilent 1100 Series System) equipped with a phenylic column (Agilent ZORBAX SB-Phenyl, 5 µm, 250 × 4.6 mm), using a mobile phase consisting of 50 mM KH_2_PO_4_, pH 2.4, at a flow rate of 0.8 mL·min − 1. Absorbance at λ 210 nm was monitored by a photodiode array detector. Lactate quantification was obtained by peak area measurement compared with standard curves and normalized on cell number.

### Lactate dehydrogenase activity

Lactate dehydrogenase (LDH) activity was measured in cell lysates using a UV–vis spectrophotometer (V-750, JASCO, Japan) equipped with thermostatic control and cuvette stirring device. Cell lysate (20 µg) was added in a 1 ml quartz cuvette containing phosphate buffer 100 mM, pyruvate 1 mM, and NADH 80 µM (λ = 340, ε = 6.22 mmol^−1^ cm^−1^). Absorbance decrease was followed for at least 60 s after cell lysate addition.

### Lipid quantification assay

The intracellular lipid droplets were visualized by staining the cells with the Nile Red fluorescent probe (ThermoFisher Scientific) as described in Rizzardi et al. [35]. Briefly, cells were seeded onto a coverslip placed in a 6-well plate at a density of 0.1 × 10^6^ cells/well. For metabolic stress conditions, cells were grown for 24 h in complete medium supplemented with 10 mM of 2-DG (Sigma Aldrich) or complete medium without glucose. Subsequently, cells were washed and fixed with 4% paraformaldehyde for 1 h at RT. Cells were rinsed twice with glycine 50 mM in PBS. Lipid droplets were stained with 1 ng/ml of Nile Red in PBS for 10 min in the dark. Cells were washed with bidistilled water and coverslips mounted in a glycerol based mounting medium on a microscope slide. Images were acquired using a Nikon C1si confocal microscope; 30 randomly chosen fields for each condition were analyzed by ImageJ software standard tool (ImageJ, National Institutes of Health).

### Radical oxygen species and mitochondrial anion superoxide measurement

Radical Oxygen Species (ROS) detection was assessed using the fluorescent probe H_2_DCFDA (2',7’-dichlorodihydrofluorescein diacetate; ThermoFisher Scientific). Cells were seeded in a 96-well plate (OptiPlate Black; PerkinElmer) at 0.2 × 10^5^ cells/well density in complete medium. After 24 h, cells were incubated for 30 min with 10 µM H_2_DCFDA dissolved in RPMI. Cells were rinsed twice with HBSS and fluorescence emission from each well was measured (λ_ex_ 485 nm; λ_em_ 535 nm) using a multiplate reader (EnSpire; PerkinElmer). The mitochondrial anion superoxide production was determined by using MitoSOX™ Red (Molecular Probes). Briefly, cells were seeded as above in 96-well plates (OptiPlate Black; Perkin Elmer). Cells were incubated with 5 µM of MitoSOX™ Red for 30 min in complete medium. After this time, cells were washed with HBSS, and the fluorescence emission was measured (λ_ex_ = 510 nm; λ_em_ = 580 nm) with a multiplate reader. For both experiments, fluorescence emission was normalized on protein content determined by Lowry’s assay.

### Glutathione levels analysis

Glutathione levels were assessed using the GSH/GSSG-Glo™ kit (Promega) following manufacturer’s instructions. Luminescence was determined using a multiplate reader (EnSpire; PerkinElmer). Data are indicated as mean ± standard error of at least 3 independent experiments.

### Calcium levels determination

Intracellular calcium was measured using the cell permeable calcium sensor Fluo-3 AM (Calcium Indicator fluorescence probe (λ_exc_ = 506 nm, λ_em_ = 526 nm; Thermo Fisher). Cells were seeded in µ-8 wells (Ibidi) and incubated overnight to allow adhesion. The day after, cells were stained with 1.5 µM of Fluo-3 AM for 30 min. Then, cells were rinsed twice with HBSS and images were acquired using a Nikon C1si (Nikon). Fluorescence intensity for each cell was calculated with ImageJ software standard tools (ImageJ, National Institutes of Health).

### Statistical analysis

Differences between two or more groups were analyzed using unpaired Student’s t-test or ANOVA. Tukey’s post hoc test was used for comparisons among groups after ANOVA analysis. Pearson’s correlation coefficient was used to investigate relationships between two variables. Reported p-values were two-sided. Statistical calculations were executed using SPSS version 20.0 (SPSS inc) and GraphPad software version 8.0 (Dotmatics). * *p* < 0.05, ** *p* < 0.01, *** *p* < 0.001, **** *p* < 0.0001.

## Results

### G6pc is downregulated in HCC and associates with poor prognosis

We previously reported the activation of mTOR and hypoxia-inducible factor (*HIF1A*) in miR-494-overexpressing cells [20]. Due to the well-known role of these pathways in metabolic reprogramming of cancer cells, here we investigated the influence of miR-494 on cell metabolism of HCC. To this aim, we interrogated Targetscan algorithm looking for miR-494 putative targets. A subsequent cross analysis was conducted to identify common target genes with a role in hepatocarcinogenesis among TargetScan gene list, and genes downregulated in human (TCGA dataset) and rat (DEN-HCC model) HCCs in comparison to surrounding livers. The Venn diagram showed 144 overlapping genes between miR-494 putative targets and downregulated mRNAs in human and rat HCCs (Fig. S1A, Supplementary Table 6). Gene ontology (GO) analysis of the cellular component (CC) revealed a significant enrichment of genes from the mitochondrial matrix (GO:0005759) while the analysis of biologic processes (BP) showed a significant enrichment of multiple metabolic processes involving lipids (GO:0070328, GO:0042632, GO:000661) and amino acids (Fig. S1A, Supplementary Material). Reactome pathway analysis also identified multiple genes directly implicated in metabolic pathways (R-HSA-1430728). Intriguingly, among this 48-metabolic pathway gene list, G6pc resulted among the top downregulated genes in DEN-HCC rats (Supplementary Table 7). Since G6pc plays a central role in liver metabolism but its contribution to hepatocarcinogenesis is poorly understood, we decided to pinpoint its biologic functions in metabolic plasticity of HCC cells unveiling its relationship with miR-494.

**Fig. 1:**
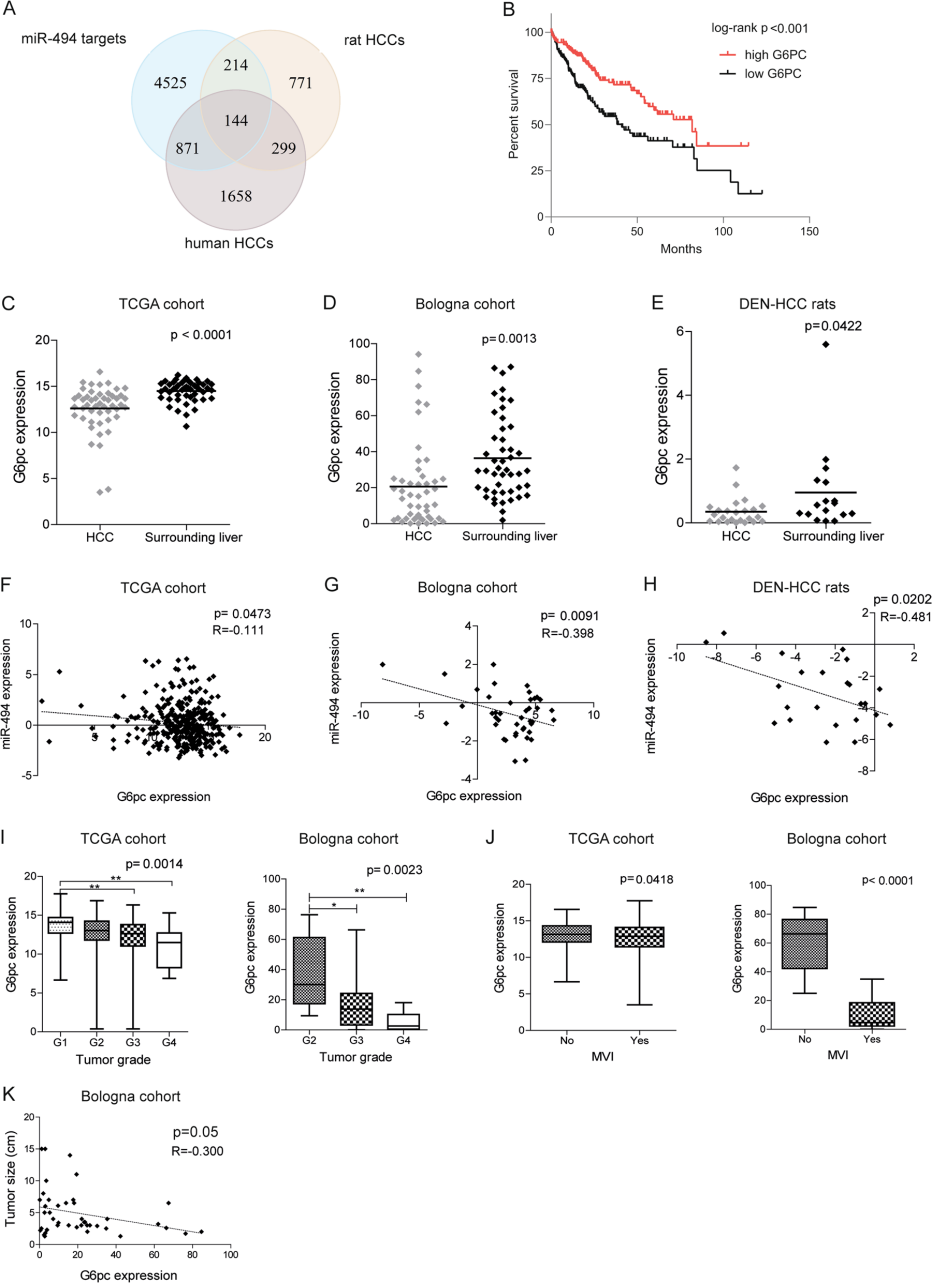
Deregulated expression of G6pc in human and rat HCCs and association with clinicopathological features. **A** Venn diagram of miR-494 putative targets (Targetscan algorithm) and downregulated genes in human (TCGA-HCC) and rat (DEN-HCC model) HCCs with respect to surrounding livers. **B** Kaplan–Meier curves of high and low G6pc-expressing HCCs (TCGA cohort). **C-E** Box plot graphs of G6pc mRNA levels in HCC and surrounding livers from the TCGA (*N* = 49) and Bologna (*N* = 46) cohorts and DEN-HCC rats (*N* = 18). Y-axes report G6pc mRNA expression. Real Time PCR was run in triplicate. **F–H** Correlation graphs between miR-494 and G6pc mRNA levels in HCC tissues of the TCGA (*N* = 319) and Bologna (*N* = 42) cohorts and tumor nodules (*N* = 23) of DEN-HCC rats. Axes report 2^−ΔΔCt^ values corresponding to miR-494 and G6pc levels transformed in a log2 form. Real Time PCR was run in triplicate. **I** Box plot graphs of G6pc mRNA levels in HCCs from the TCGA (*N* = 365) and Bologna (*N* = 42) cohorts according to tumor grade. On the top of each graph is reported the p-value relative to ANOVA, whereas stars represent comparison between groups (Tukey’s post hoc test). Y-axes report G6pc mRNA expression. Real Time PCR was run in triplicate. **J** Box plot graphs of G6pc mRNA levels in HCCs of the TCGA (*N* = 298) and Bologna (*N* = 25) cohorts divided according to the presence or absence of microvascular invasion (MVI). Y-axes report G6pc mRNA expression. Real Time PCR was run in triplicate. **K** Correlation graph between G6pc mRNA levels and tumor size of HCC patients (*N* = 42) from the Bologna cohort. Axes report 2^−ΔΔ^.^Ct^ values corresponding to G6pc mRNA levels and tumor size (cm). GAPDH was used has housekeeping gene. Real Time PCR was run in triplicate. ANOVA, two-tailed unpaired Student's t-test and Pearson’s correlation were used. * *P* ≤ 0.05; ** *P* ≤ 0.01

Interestingly, lower G6pc levels associated with decreased overall survival in HCC patients (Fig. 1B). In addition, a downregulation of *G6PC* mRNA was detected in tumor specimens with respect to surrounding livers in two HCC patient cohorts and in tumors from DEN-HCC rats (Fig. 1C-E). Cases were categorized as high or low expressing miR-494 based on our previous observation that approximately 25% of HCCs upregulated miR-494 [20] which was confirmed in the TCGA-cohort. A differential expression analysis in the TCGA-HCC dataset revealed a downregulation of G6pc in the high miR-494 expressing group with respect to the low one (fold change = 1.8; *p* = 0.045). In agreement, an inverse correlation between G6pc and miR-494 expression was identified in human and rat HCCs (Fig. 1F-H) but not in surrounding livers (Fig. S1B-D), suggesting the pivotal role of miR-494 in mediating G6pc regulation in liver tumors. The molecular variability of human HCCs, characterized by intra-tumor heterogeneity and multiple etiopathogenesis, might explain the low degree of correlation observed in the TCGA dataset. In line, a better correlation is reported in the Bologna cohort where most of patients (67%) are HCV-positive and in the animal model. Moreover, besides the fine regulation of miRNAs, other factors regulate the expression of target genes. In this regard, the transcription factor HIF-1A which expression is stabilized by miR-494 [20] contributes to the transcriptional activation of G6pc itself [36]. We therefore investigated if a correlation could exist between miR-494 or G6pc and HIF-1A transcriptional metabolic targets ALDOA and GLUT1 in HCC patients. Interestingly, a positive correlation between miR-494 and ALDOA or GLUT1 mRNAs (even though not statistically significant for GLUT1) and a negative correlation between G6pc and HIF-1A transcriptional targets were detected in HCC specimens (Fig. S2A-E). These findings suggest that miR-494 prevails over HIF-1A in the regulation of G6pc expression and highlight the complex network of miRNA-mediated effects concurring to the metabolic phenotype of liver tumors.

Clinicopathological associations showed that G6pc expression is lower in high-grade tumors and in patients with microvascular invasion (MVI) and negatively correlates with tumor size (Fig. 1I-K). On the contrary, HIF-1A targets resulted upregulated in HCC (even though not statistically significant for GLUT1) but showed no significant associations with clinicopathological features except for high ALDOA expression in patients with MVI (Fig. S2F-L). Because of G6pc association with aggressive tumor characteristics, we investigated molecular mechanisms leading to its deregulation in HCC.

### MiR-494 targets G6pc in HCC

**Fig. 2:**
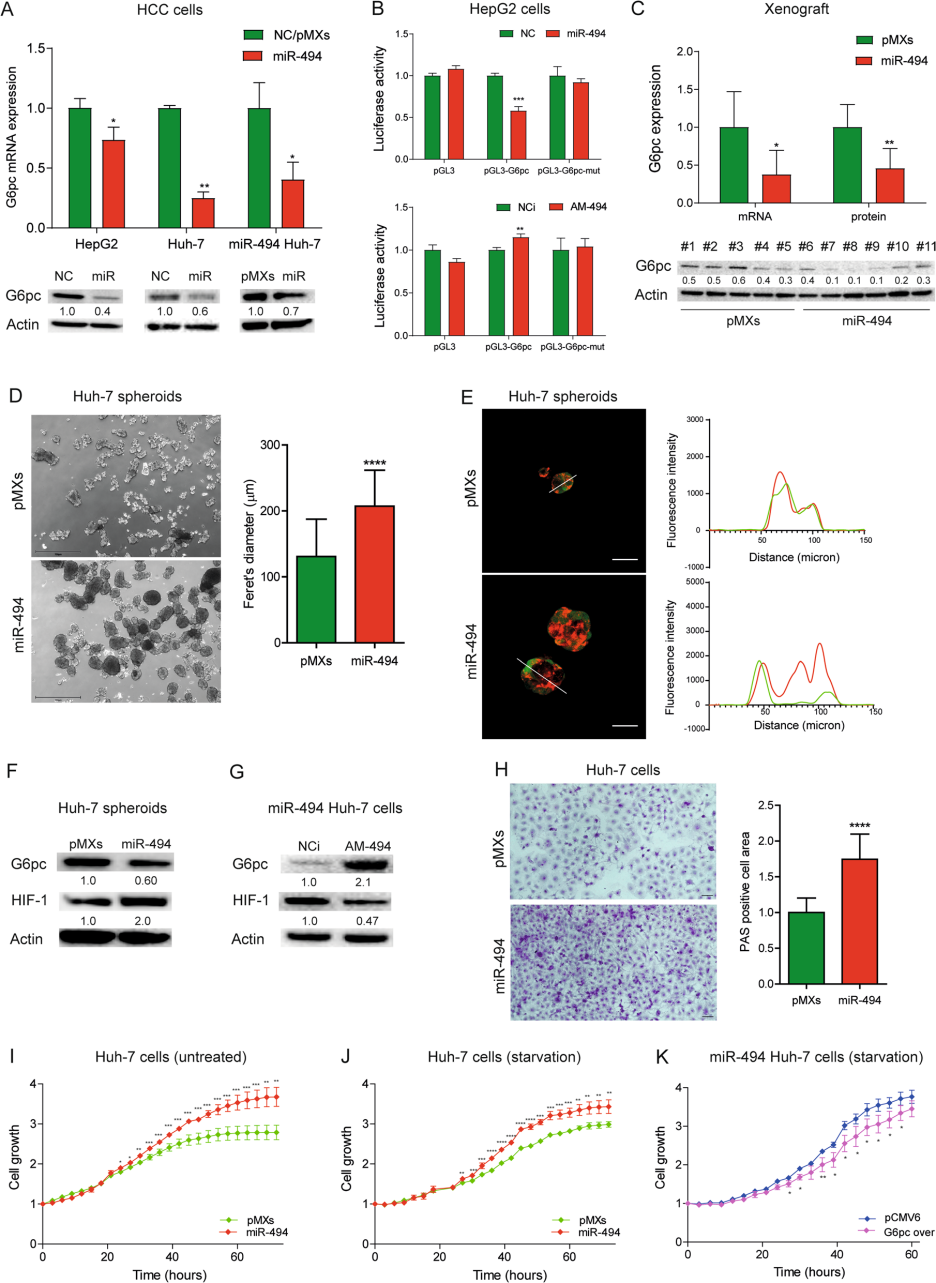
G6pc is a target of miR-494 in HCC. **A** Real Time PCR and WB analyses of G6pc expression following miR-494 overexpression in transfected HCC cells and infected Huh-7 cells (miR-494 Huh-7). NC: negative control precursor miRNA. PMXs: control vector. Y-axis reports 2^−ΔΔ^.^Ct^ values corresponding to G6pc mRNA levels normalized to controls (NC or pMXs). Mean ± SD values are displayed. Beta-actin was used as housekeeping gene for Real Time and WB experiments. Real Time PCR analysis was performed in two independent experiments in triplicate; WB analysis was performed in two independent experiments. **B** Dual-luciferase activity assay of wild type and mutant (mut) G6pc-3UTR vectors (pGL3-G6pc) co-transfected with miR-494 or anti-miR-494 (AM-494) in HepG2 cells. NC: negative control precursor miRNA. NCi: negative inhibitor control miRNA. Y-axes report the firefly/renilla ratio normalized to controls (NC or NCi). Mean ± SD values are displayed. Analysis was performed in two independent experiments in triplicate. **C** Real Time PCR and WB analyses of G6pc expression in tumor masses (N = 16) of xenograft mice obtained following subcutaneous injection of miR-494-overexpressing and control (pMXs) Huh-7 cells. Y-axis reports G6pc mRNA and protein levels normalized to control. Mean ± SD values are displayed. Beta-actin was used as housekeeping gene for Real Time and WB experiments. Real Time PCR was run in triplicate. **D** Representative images (4X magnification) of miR-494-overexpressing and control (pMXs) Huh-7 spheroids at 24 h. Data were obtained by measuring feret’s diameter (µm) of thirty randomly selected spheroids in two independent experiments. Mean ± SD values are displayed. Scale bars, 750 μm. **E** Representative confocal images of control (pMXs) and miR-494-overexpressing Huh-7 spheroids expressing GFP (green signal) and stained with the live-cell oxygen sensor BTP (red signal), which fluorescence emission is quenched by molecular oxygen. Traces represent red and green fluorescence intensity along the linear regions of interest traced in the merged images to visualize signal distribution. At least ten spheroids for condition were analyzed in two independent experiments. Scale bars, 25 μm. **F** WB analysis of G6pc and HIF-1 in Huh-7 spheroids and (**G**) miR-494-overexpressing Huh-7 cells following AM-494 transfection. NCi: negative inhibitor miRNA control. PMXs: control vector. Beta-actin was used as housekeeping gene. WB analysis was performed in two independent experiments. **H** Representative images (20X magnification) of PAS staining in control (pMXs) and miR-494-overexpressing Huh-7 cells. Y-axis reports the percent of PAS positive cell area normalized to control. Mean ± SD values are displayed. Five randomly selected fields were analyzed from three independent experiments. Scale bars, 20 μm. **I**, **J** Growth curves of control (pMXs) and miR-494-overexpressing Huh-7 cells in standard and starved culture conditions. Growth curves were normalized to T0. Mean ± SD values are reported. Two independent experiments were performed in quadruplicate. **K** Growth curves of miR-494-overexpressing Huh-7 cells transfected with G6pc overexpressing (G6pc over) or control (pCMV6) vector and grown in starved (medium without FBS) culture conditions. Growth curves were normalized to T0. Mean ± SD values are reported. Two independent experiments were performed in quadruplicate. Statistical significance was determined by two-tailed unpaired Student's t-test. *P ≤ 0.05; **P ≤ 0.01; ***P ≤ 0.001; ****P ≤ 0.0001

Bioinformatics analysis identified one poorly conserved site for miR-494 in the 3’-untranslated region (3’UTR) of G6pc mRNA (Fig. S3A). To demonstrate the regulation of G6pc by miR-494, we performed functional analysis and luciferase reporter assay in HCC cells. MiR-494 was overexpressed and silenced in HepG2 and Huh-7 cell lines showing low miR-494 basal levels [20] in the presence of intermediate/high G6pc protein expression (Fig. S3B). MiR-494 overexpression decreased G6pc mRNA and protein levels in both transiently transfected HepG2 and Huh-7 cells and stably infected miR-494-overexpressing Huh-7 cells, while miR-494 silencing determined the opposite in the same cell lines (Fig. 2A, S3C). MiR-494 co-transfection in HepG2 cells decreased the luciferase activity of G6pc-3’UTR vector, whereas anti-miR-494 (AM-494) caused the opposite. No significant variations were observed when G6pc-3'UTR mutated vector was considered (Fig. 2B). To confirm in vitro data, G6pc expression was assayed in xenograft mice obtained from miR-494-overexpressing and control (pMXs) Huh-7 cells. Real Time PCR and WB analyses showed reduced G6pc mRNA and protein levels in tumors from miR-494-overexpressing cells with respect to controls (Fig. 2C). These experiments demonstrated that miR-494 directly modulates G6pc expression in HCC preclinical models, leading to mRNA degradation.

To deepen our knowledge regarding the tumorigenic potential of miR-494/G6pc axis in HCC, we employed a 3D in vitro model. MiR-494 overexpression in Huh-7 cells decreased the time of spheroids formation and increased their size as observed at 24 and 48 h (Fig. 2D, S3D). Spheroids formation was not valuable in HepG2 cells due to their low spheroidogenic capacity (Fig. S3E). To evaluate G6pc contribution to miR-494-mediated phenotype, we performed rescue assays by transfecting G6pc overexpression vector in miR-494-overexpressing Huh-7 cells. Interestingly, rescue experiments with G6pc overexpression vector in miR-494 overexpressing Huh-7 cells, but not in control cells, reverted their 3D phenotype decreasing their size, and activating the apoptotic cascade (Fig. S3F-H), demonstrating that G6pc targeting mediates the biologic effects of miR-494 on 3D cell growth in HCC. Spheroids from miRNA-overexpressing cells stained with the live-cell oxygen sensor BTP displayed higher red fluorescence compared with controls, which accounts for a more hypoxic state possibly due to their larger dimensions (Fig. 2E). We confirmed G6pc downregulation also in miR-494-derived spheroids. Noticeably, in 3D and 2D-grown HCC cells, miR-494 induced HIF-1A stabilization suggesting a metabolic advantage in large–size 3D structure formations (Fig. 2F, S3I). In line, AM-494 transfection decreased HIF-1A protein levels in HepG2 cells and miRNA-overexpressing Huh-7 cells (Fig. 2G, S3I). To evaluate the contribution of HIF-1A stabilization to miR-494-dependent 3D cell growth, we silenced HIF-1A by using three Dicer-substrate short interfering RNAs (DsiRNAs) in miR-494-overxepressing Huh-7 cells (Fig. S3J). DsiRNA C was chosen because it showed the best silencing efficacy at both mRNA and protein level. HIF-1A silencing in miR-494-overexpressing Huh-7 cells affected 3D cell growth by decreasing their size (Fig. S3K), proving HIF-1A contribution, together with G6pc targeting, to miR-494-mediated 3D phenotype.

We investigated if the increase of glucose-6-phosphate levels could lead to glycogen accumulation in miR-494-overexpressing cells; to this end, we quantified intracellular glycogen content by PAS staining in in vitro models. As expected, miR-494-overexpressing Huh-7 and HepG2 cells displayed a more abundant glycogen staining, while the transfection of G6pc overexpression vector in miRNA-overexpressing Huh-7 cells reduced glycogen staining with respect to controls (Fig. 2H, S4A, B). Similarly, HIF-1A silencing in miR-494-overexpressing Huh-7 cells caused a decrease (even though not statistically significant) of glycogen staining (Fig. S4C), highlighting HIF-1A contribution to the metabolic plasticity of miR-494-overexpressing HCC cells. PAS staining was performed also in a preliminary series of FFPE specimens from xenograft mice and HCC patients showing a slight increase in glycogen staining, even though not statistically significant, in tumor hepatocytes from the high miR-494 expressing group (Fig. S4D, E).

To assess if miR-494 could influence the proliferation of HCC cells, we used a live-cell imaging system that allows the monitoring of cell growth over time in controlled culture conditions. MiR-494-overexpressing Huh-7 cells showed a decreased doubling time in standard (24.2 vs 32.8 h) and serum-deprived (23.5 vs 32.1 h) culture media compared to controls (Fig. 2I, J). Interestingly, G6pc overexpression slowed down the cell growth of miR-494-overexpressing Huh-7 cells when cultured in serum-deprived medium but not in standard growing conditions (Fig. 2K, S4F), suggesting that its targeting by miR-494 confers metabolic advantages specifically in harsh growth settings. The dilution of G6pc overexpression vector in proliferating cells might be the cause of the lack of statistical significance observed at the last time point (Fig. 2K).

These findings demonstrated the involvement of miR-494/G6pc axis and HIF-1A activation in proliferation, spherogenicity, and glycogen accumulation in HCC cells.

### MiR-494 regulates cellular metabolism in HCC cells

**Fig. 3:**
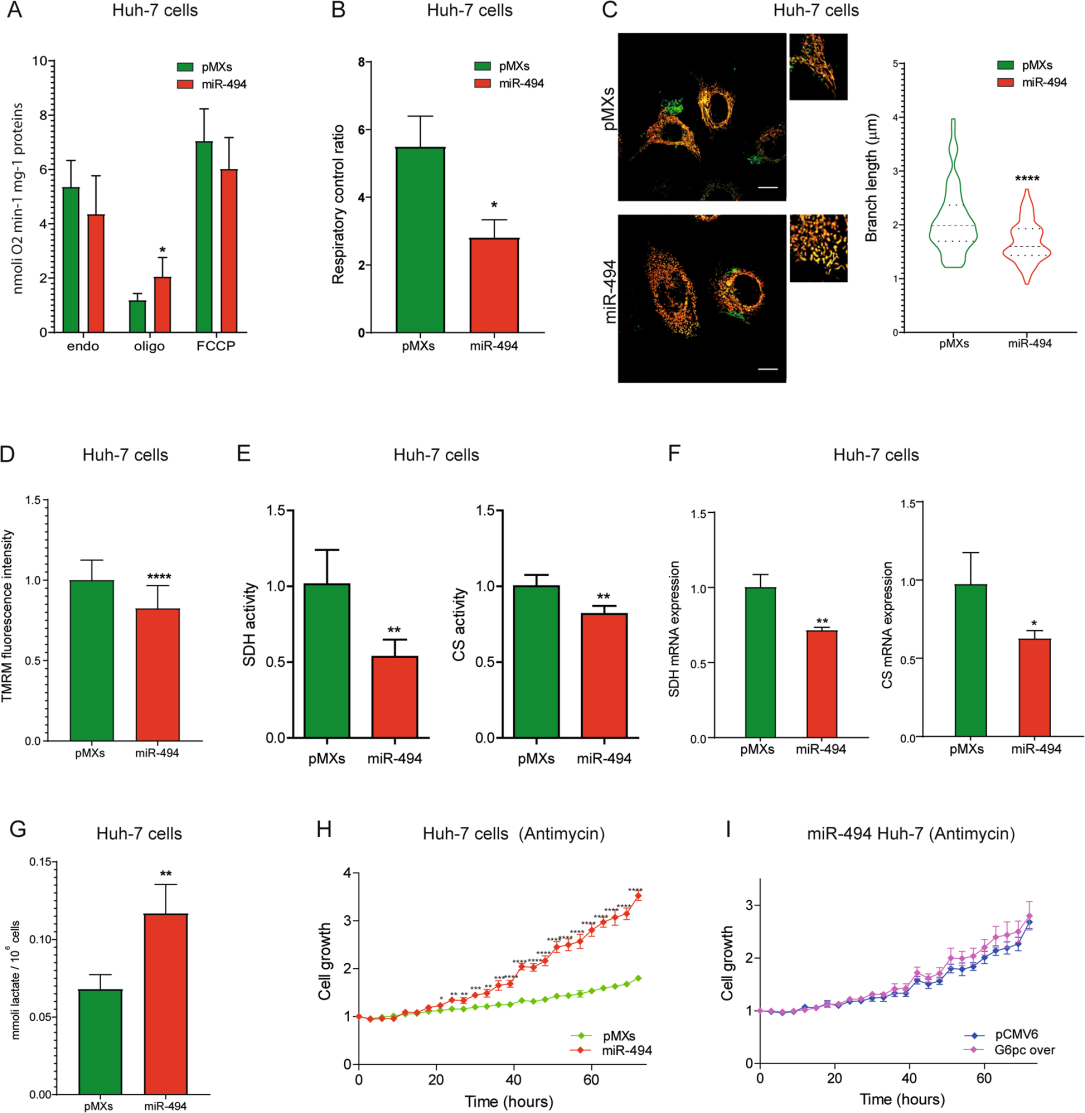
MiR-494 regulates cellular metabolism in HCC cells. **A** Oxygen consumption rate in control (pMXs) and miR-494-overexpressing Huh-7 cells measured in standard medium (endogenous respiration); in the presence of oligomycin A (Oligo) and carbonyl cyanide 4-(trifluoromethoxy) phenylhydrazone (FCCP). Mean ± SD values are displayed. Three independent experiments were performed. **B** Respiratory control ratio of control (pMXS) and miR-494-overexpressing Huh-7 cells. Three independent experiments were performed. **C** Representative confocal images of control (pMXs) and miR-494-overexpressing Huh-7 cells after staining with TMRM (red) and Mitotracker Green (MTG, green). Inset panels show magnification of selected areas and the violin plot refers to the quantification of the mean length of mitochondrial branches (µM). Data were obtained by measuring at least thirty randomly selected cells in two independent experiments. **D** Quantification of ΔΨm in control (pMXs) and miR-494-overexpressing Huh-7 cells. Data were obtained by measuring five randomly selected fields in three independent experiments and are expressed as TMRM signal intensity normalized to control. Mean ± SD values are displayed. **E** Enzymatic activity of succinate dehydrogenase (SDH) and citrate synthase (CS) in control (pMXs) and miR-494-overexpressing Huh-7 cells. The Y-axis reports the enzymatic activity (µmol*min^−1^*mg^−1^) normalized to control. Mean ± SD values are displayed. Three independent experiments were analyzed in duplicate. **F** Real Time PCR analysis of succinate dehydrogenase (SDH) and citrate synthase (CS) expression in control (pMXs) and miR-494 overexpressing Huh-7 cells. Y-axis reports 2^−ΔΔCt^ values corresponding to mRNA levels normalized to control. Mean ± SD values are displayed. Beta-actin was used as housekeeping gene. Real Time PCR analysis was performed in two independent experiments in triplicate. **G** Extracellular lactate quantification by HPLC in control (pMXs) and miR-494 overexpressing Huh-7 cells. Mean ± SD values are displayed. Three independent experiments were analyzed in duplicate. **H** Growth curves of control (pMXs) and miR-494 overexpressing Huh-7 cells in the presence of Antimycin A. Growth curves were normalized to T0. Mean ± SD values are reported. **I** Growth curves of miR-494 overexpressing Huh-7 cells transfected with G6pc overexpressing or empty (pCMV6) vector in the presence of Antimycin A. Growth curves were normalized to T0. Mean ± SD values are reported. **H**, **I** Live imaging curves were performed in two independent experiments in quadruplicate. Statistical significance was determined by two-tailed unpaired Student's t-test. * *P* ≤ 0.05; ** P ≤ 0.01; ***P ≤ 0.001; ****P ≤ 0.0001. PMXs: empty vector

To investigate the role of miR-494 overexpression on cellular metabolism, we measured mitochondrial oxygen consumption rate (OCR) in intact cells under basal conditions (basal respiration) and in the presence of oligomycin A, which inhibits ATPase activity, and FCCP, which dissipates the mitochondrial potential allowing the maximal oxygen consumption rate (uncoupled respiration). Compared to controls, miR-494-overexpressing Huh-7 cells showed increased OCR in the presence of oligomycin A and decreased, even though not significantly, uncoupled respiration (Fig. 3A). Oxygen consumption analysis displayed that miR-494 cells have lower respiratory control index (RCI) and a trend toward reduced ATP-linked respiration (Fig. 3B, S5A). Similarly, miR-494-overexpression in HepG2 cells increased OCR in the presence of oligomycin A and decreased the RCI (Fig. S5B, C). Notably, rescue experiments with G6pc overexpression vector showed an increase of both basal OCR and ATP-linked respiration in miR-494-overexpressing Huh-7 cells, but not in control cells, reverting their glycolytic metabolism towards OXPHOS respiration, demonstrating miR-494/G6pc axis involvement in metabolic reprogramming of HCC cells (Fig. S5D-G).

Since a low respiratory control ratio usually indicates mitochondrial dysfunction, we analyzed the mitochondrial status by assessing the mitochondrial network organization using MitoTracker green (Fig. 3C, left panel). Morphological analysis was performed by measuring mitochondrial branch length, which is a descriptive parameter of network organization. Cells overexpressing miR-494 showed a fragmented network (Fig. 3C, right panel), consistent with lower OXPHOS activity. In agreement with this finding, the quantification of mitochondrial transmembrane potential (ΔѰ) using TMRM fluorescent dye showed lower ΔѰ levels in miR-494 cells compared to controls (Fig. 3D). Moreover, we measured the specific activity of two tricarboxylic acid cycle enzymes: Succinate Dehydrogenase (SDH) and citrate synthase (CS). We found a decreased activity and mRNA expression of SDH and CS in miR-494-overexpressing HCC cells (Fig. 3E, F, S5H, S5I). Conversely, G6pc overexpression in miR-494-overexpressing Huh-7 cells, but not in control cells, increased both the activity and mRNA levels of these two enzymes (Fig. S5J, K). Interestingly, CS is a miR-494 hypothetic target but, differently from G6pc, it is upregulated in HCC and does not correlate with patient survival (Fig. S5L-N), therefore we did not investigate it further. To investigate the contribution of HIF-1A pathway, HIF-1A was silenced in miR-494-overexpressing Huh-7 cells showing no variations in OCR and ATP-linked respiration, even if SDH and CS activities were increased (not statistically significant) in the same setting (Fig. S6A-C). Taken together, these findings suggest a stronger influence of G6pc targeting over HIF-1A activation on miR-494-mediated metabolic shift of HCC cells.

To assess the dependence of miR-494-overexpressing cells on anaerobic glucose metabolism, we determined lactate levels in the extracellular medium by HPLC. We found increased levels of extracellular lactate in miR-494-overexpressing HCC cells (Fig. 3G, S6D), mirrored by a trend towards increased lactate dehydrogenase (LDH) activity (Fig. S6E), indicating a more glycolytic metabolism. To investigate this point, we followed the proliferation of miR-494-overexpressing Huh-7 cells in the presence of antimycin A (AA), which forces cells to use glycolysis for energy supply by blocking the mitochondrial respiratory chain activity. The cell growth of miR-494 cells was less affected by AA treatment, confirming that they can efficiently rely on glycolysis when the oxidative phosphorylation system is affected (Fig. 3H). Notably, G6pc overexpression in miR-494-overexpressing cells did not recover AA sensitivity (Fig. 3I), suggesting that miR-494 may act on multiple targets in this setting such as by activating the HIF1A metabolic pathway (Fig. S6F). On the contrary, HIF-1A silencing in miR-494-overexpressing Huh-7 cells did not change lactate production, highlighting the complexity of miRNA modulation in metabolic reprogramming of liver tumors (Fig. S6G). In agreement with in vitro findings, data envelopment analysis (DEA) conducted between high and low miR-494 expressing tumors from the TCGA-HCC cohort showed a significant downregulation of multiple OXPHOS genes (Supplementary Table 8). These findings highlighted the profound effect that miR-494 exerts on HCC cell metabolism, resulting in a metabolic shift to aerobic glycolysis.

### MiR-494 regulates lipid metabolism in HCC cells

**Fig. 4:**
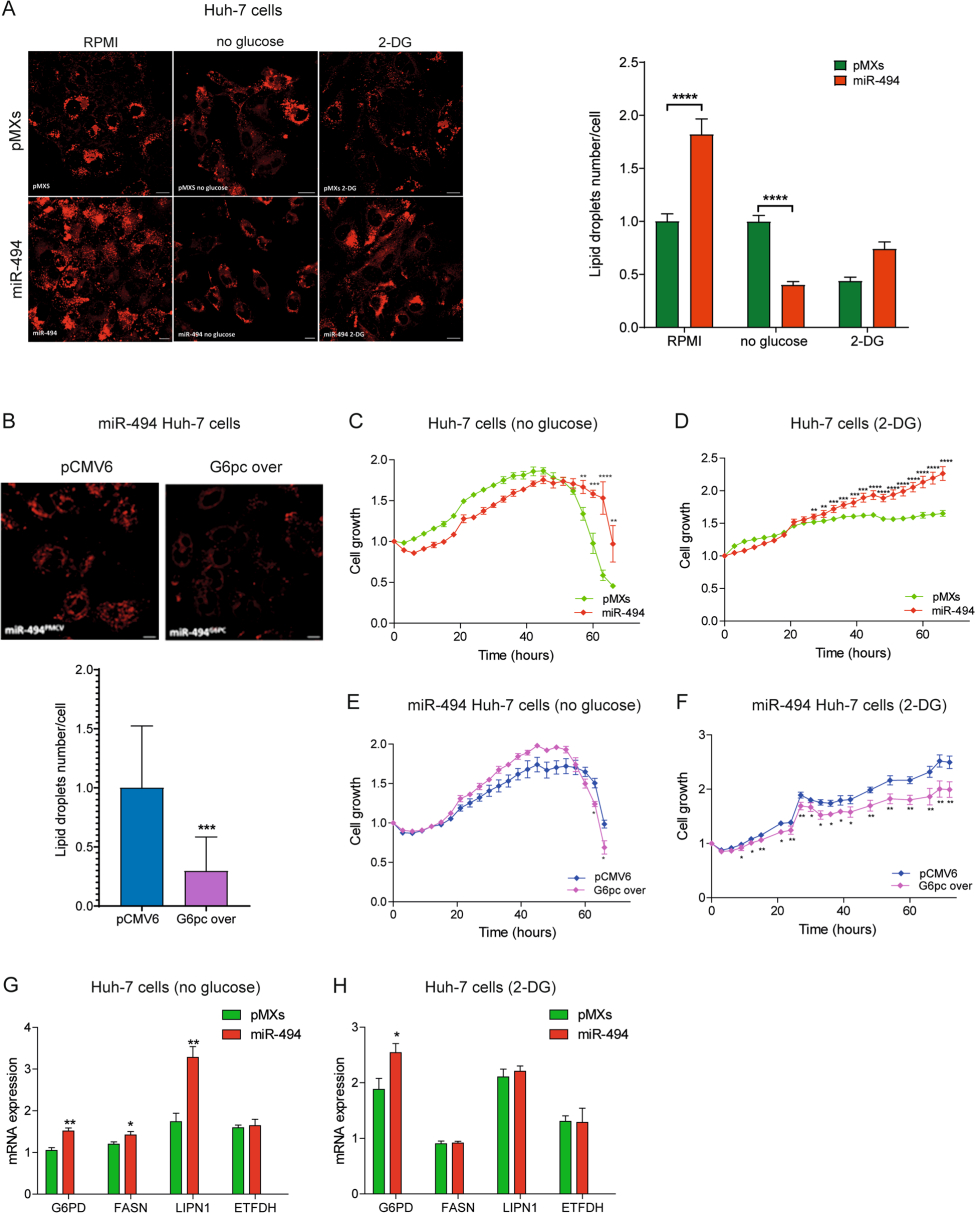
MiR-494 regulates lipid metabolism in HCC cells. **A** Representative confocal images of control (pMXs) and miR-494-overexpressing Huh-7 cells stained with Nile Red to visualize lipid droplets (LDs) accumulation. The cells were cultured for 24 h under standard conditions (RPMI), in medium without glucose, and in the presence of 2-deoxy glucose (2-DG). The Y-axis shows the quantification of LDs number per cell in each condition normalized to pMXs cells cultured in standard conditions. Mean ± SD values are reported. Two independent experiments were performed. Scale bar = 20 µm. **B** Representative confocal images of miR-494-overexpressing Huh-7 cells following transfection with G6pc overexpressing (G6pc over) or control (pCMV6) vectors for 24 h and stained with Nile Red. The column bar graph below shows the quantification of LDs number per cell respect normalized to control. Two independent experiments were performed. Scale bar, 20 µm. **C** Growth curves of control (pMXs) and miR-494-overexpressing Huh-7 cells cultured in no-glucose conditions or (**D**) in the presence of 2-DG. Growth curves were normalized to T0. Mean ± SD values are reported. The experiments were performed in two independent experiments in quadruplicate. **E** Growth curves of miR-494-overexpressing Huh-7 cells transfected with G6p-overexpressing (G6pc over) or control (pCMV6) vector and cultured in no glucose conditions or (**F**) in the presence of 2-DG. Growth curves were normalized to T0. Mean ± SD values are reported. The experiments were performed in two independent experiments in quadruplicate. **G** Real time PCR analysis of metabolic genes involved in lipid metabolism and pentose phosphate pathway in control (pMXs) and miR-494-overexpressing Huh-7 cells grown for 24 h in no glucose medium or (**H**) in the presence of 2-DG. Y-axis reports 2^−ΔΔ^.^Ct^ values corresponding to mRNA levels. Mean ± SD values are displayed. Beta-actin was used as housekeeping gene. Real Time PCR analysis was performed in two independent experiments in triplicate. The statistical analysis was performed using two-tailed unpaired Student's t-test. * *P* ≤ 0.05; ** P ≤ 0.01; *** P ≤ 0.001; **** *P* ≤ 0.0001

Since G6pc deletion is responsible for lipid accumulation in the liver of experimental animal models [37], here we evaluated whether an altered lipid metabolism could be observed in miR-494-overexpressing cells as well. To this end, we investigated the lipid droplets accumulation in miRNA and control Huh-7 cells using the fluorescent Nile Red dye, which stains intracellular lipid droplets (LDs). The confocal microscope analysis evidenced a significant increase in LDs number in miR-494-overexpressing HepG2 and Huh-7 cells (Fig. 4A, S7A). Interestingly, G6pc overexpression but not HIF-1A silencing dropped down the intracellular lipid content in miR-494-overexpressing Huh-7 cells (Fig. 4B, S7B) highlighting the role of G6pc in miRNA-mediated dysregulation of lipid metabolism in HCC.

Considering the role of miR-494 in glycolytic reprogramming of Huh-7 cells, we explored the growth of HCC cells bearing miR-494 enforced expression in two different conditions: (i) in the absence of glucose and (ii) in the presence of the glycolysis inhibitor 2-deoxy-glucose (2-DG). A prolonged survival of miR-494-overexpressing cells was observed in glucose-deprived medium as well as in the presence of 2-DG. As expected, G6pc overexpression in miR-494-overexpressing cells partially reverted their proliferative advantage (Fig. 4C-F). We next evaluated whether changes in lipid metabolism could support, at least in part, the resistance to metabolic stress conferred by miR-494 overexpression in Huh-7 cells. To this end, we measured the number of LDs after 24 h of incubation in glucose-deprived and 2-DG-supplemented media. Of note, a different behavior in lipid consumption between miR-494-overexpressing and control cells was observed in glucose-deprived medium only, suggesting lipid catabolism as a discriminating factor allowing the survival of miR-494-overexpressing cells in this condition (Fig. 4A). Similarly, a lower amount of glycogen was detected in miR-494-overexpressing cells when cultured in glucose-free medium with respect to standard medium (Fig. S7C).

To detail further the metabolic pathways involved in cell survival, we compared the expression of a set of metabolic-relevant genes (*G6PD*, *FASN*, *LPIN1*, *ETFDH*) in control and miR-494-overexpressing cells cultured in metabolic stress conditions. In line with lipid consumption, the largest changes in gene expression were detected in miR-494-overexpressing cells after exposure to glucose-deprived medium. Specifically, glucose deprivation increased the expression of G6PD, FASN, and LPIN1, suggesting an adaptation response. On the contrary, no variation of ETFDH mRNA was observed in these settings, letting us hypothesize that miR-494-overexpressing cells did not require an energetic boost from the β-oxidation pathway in these conditions (Fig. 4G, H).

Regarding HCC patients, a downregulation of multiple genes involved in lipid metabolism and especially in fatty acids oxidation was seen in the DEA analysis of high versus low expressing tumors from the TCGA-HCC (Supplementary Table 8). Notably, G6PD, FASN and LPIN1 are upregulated in HCC compared with non-tumor livers in both HCC cohorts (Supplementary Table 9) while ETFDH is downregulated (Fig. S7D), highlighting the likely role of lipid metabolism and PPP pathways in liver cancer. Even though not deregulated in miR-494-overexpressing Huh-7 cells under challenging conditions, an inverse correlation between miR-494 and ETFDH and a direct correlation between ETFDH and G6pc were detected in HCC patients, suggesting a possible relationship in liver tumors. These data fit well with the decreased overall survival of the HCC group with low ETFDH expression (Fig. S7E-G), further emphasizing the contribution of lipid metabolism to metabolic plasticity of HCC and its involvement in tumor aggressiveness.

### MiR-494 affects redox homeostasis in HCC cells

**Fig. 5:**
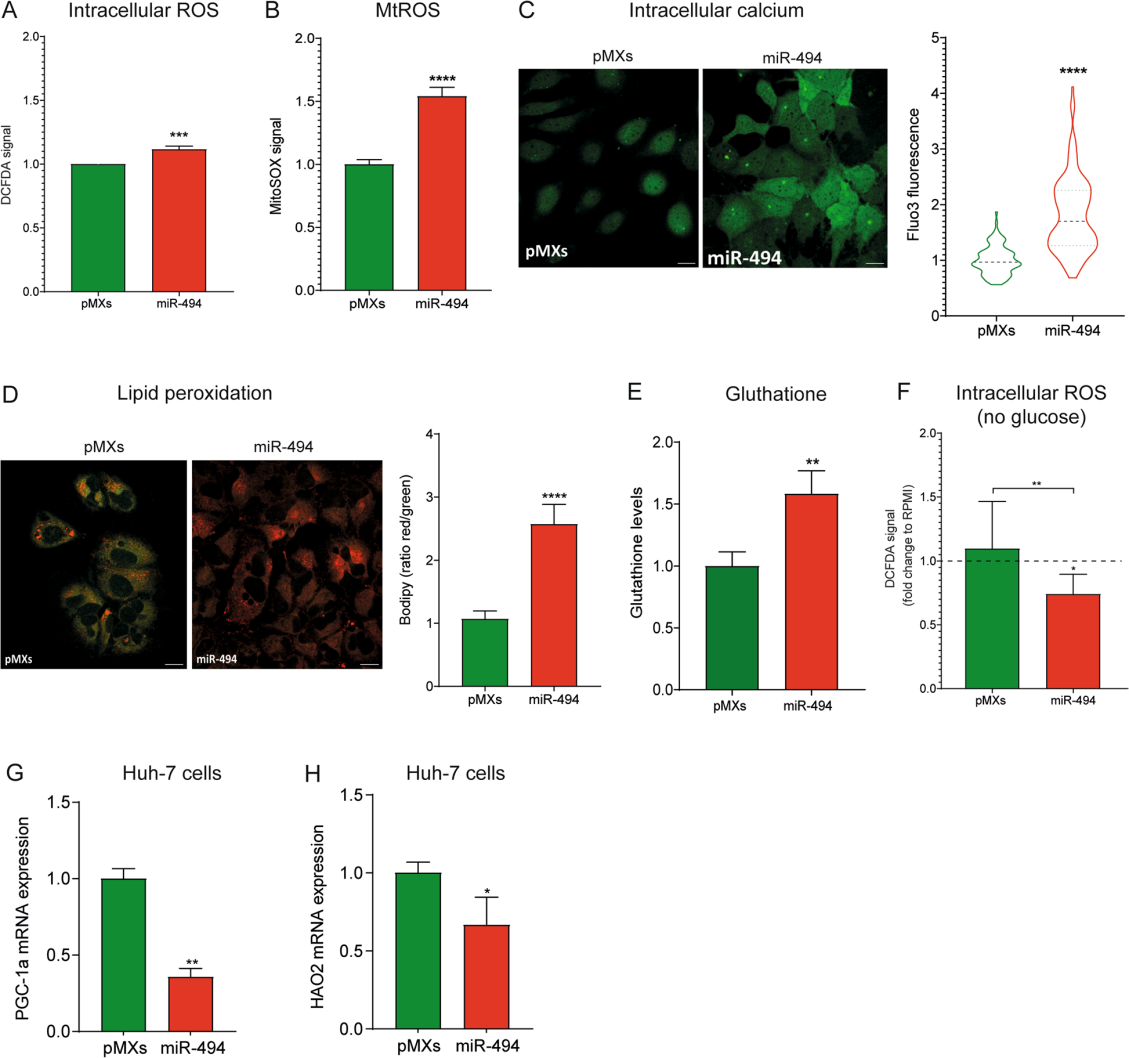
MiR-494 affects redox homeostasis in HCC cells. **A** ROS production in control (pMXs) and miR-494-overexpressing Huh-7 cells measured using dichlorofluorescin diacetate (DCFDA) and (**B**) mitochondrial superoxide production measured using the mitochondrial specific probe MitoSOX Red. Y-axes reports the arbitrary fluorescence units normalized to controls. Mean ± SD values are displayed. Three independent experiments were performed. **C** Representative confocal microscope images of control (pMXs) and miR-494-overexpressing Huh-7 cells stained with Fluo-3 AM. Violin plot reports the quantification of Fluo-3 intensity emission per cell normalized to control. Five randomly selected fields were analysed in two independent experiments. Mean ± SD values are displayed. Scale bar = 30 µm. **D** Lipid membrane peroxidation status in control (pMXs) and miR-494-overexpressing Huh-7 cells measured using the lipid peroxidation sensor BODIPY™ 581/591 C11. The oxidation of the dye results in a green-shift fluorescence emission peak. Y-axis reports quantification of the Red/Green intensity ratio per cell normalized to control. Five randomly selected fields were analysed in three independent experiments. Mean ± SD values are displayed. Scale bar, 30 µM. **E** Glutathione content in control (pMXs) and miR-494-overexpressing Huh-7 cells. Y-axis reports the luminescence signal relative to glutathione levels normalized to control. Mean ± SD values are displayed. Three independent experiments were performed. **F** ROS production in control (pMXs) and miR-494-overexpressing cells after 24 h of exposure to no-glucose medium measured using DCFDA. Data are presented as fold change of ROS production in no-glucose with respect to normal culture condition (RPMI). Three independent experiments were performed. **G** Real time PCR analysis of PGC-1alpha and (**H**) HAO2 mRNA levels in control (pMXs) and miR-494-overexpressing Huh-7 cells. Y-axis reports 2^−ΔΔ^.^Ct^ values corresponding to mRNA levels. Normalized mean ± SD values are displayed. Beta-actin was used as housekeeping gene. Real Time PCR analysis was performed in two independent experiments in triplicate. The statistical analysis was performed using two-tailed unpaired Student's t-test. * *P* ≤ 0.05; ** P ≤ 0.01; *** P ≤ 0.001; **** P ≤ 0.0001

In the light of PPP pathway activation by miR-494 during metabolic stress, we evaluated whether an alteration of the redox homeostasis could exist in miRNA-overexpressing cells. To this end, we measured the basal level of reactive oxygen species (ROS) in miR-494-overexpressing Huh-7 cells under standard growth conditions using the fluorescent probe DCFDA. Increased ROS basal levels were observed in miR-494-overexpressing cells compared to controls (Fig. 5A). To further investigate oxidative stress, we stained the cells with the fluorogenic probe Mitosox red, finding higher levels of mitochondrial superoxide anion production in miR-494-overexpressing cells (Fig. 5B). Besides, miR-494 cells displayed higher intracellular free calcium levels, measured with the fluorescent probe Fluo-3 AM (Fig. 5C). Subsequently, we treated the cells with the lipid peroxidation sensor BODIPY C11 to assess the effect of ROS on cell membrane status. Although characterized by higher basal levels of ROS production, miR-494-overexpressing cells showed a lower degree of lipid membrane peroxidation (Fig. 5D). To explain the latter finding, we assessed the glutathione levels, an endogenously synthesized antioxidant which prevents lipid membrane peroxidation. Figure 5E shows that miR-494-overexpressing Huh-7 cells exhibit higher levels of glutathione than controls, possibly helping to maintain redox homeostasis in the presence of a higher ROS production. Interestingly, miR-494-overexpressing cells maintained the redox state homeostasis even during metabolic stress showing a decrease in ROS production when grown in glucose-free medium (Fig. 5F).

Since the critical lipid-oxidation transcription factor PPARG coactivator 1 alpha (PGC-1alpha) is a miR-494 putative target, we evaluated its expression in HCC cells, detecting lower mRNA levels in miR-494 overexpressing cells (Fig. 5G). In line with previous findings in the DEN-HCC rat model [38], a downregulation of the tumor suppressor gene hydroxy acid oxidase 2 (HAO2) was also observed in miR-494-overexpressing cells (Fig. 5H), further highlighting their metabolic plasticity and ability to neutralize ROS during harsh growing conditions.

### MiR-494 represents a potential circulating biomarker and therapeutic target in HCC

**Fig. 6:**
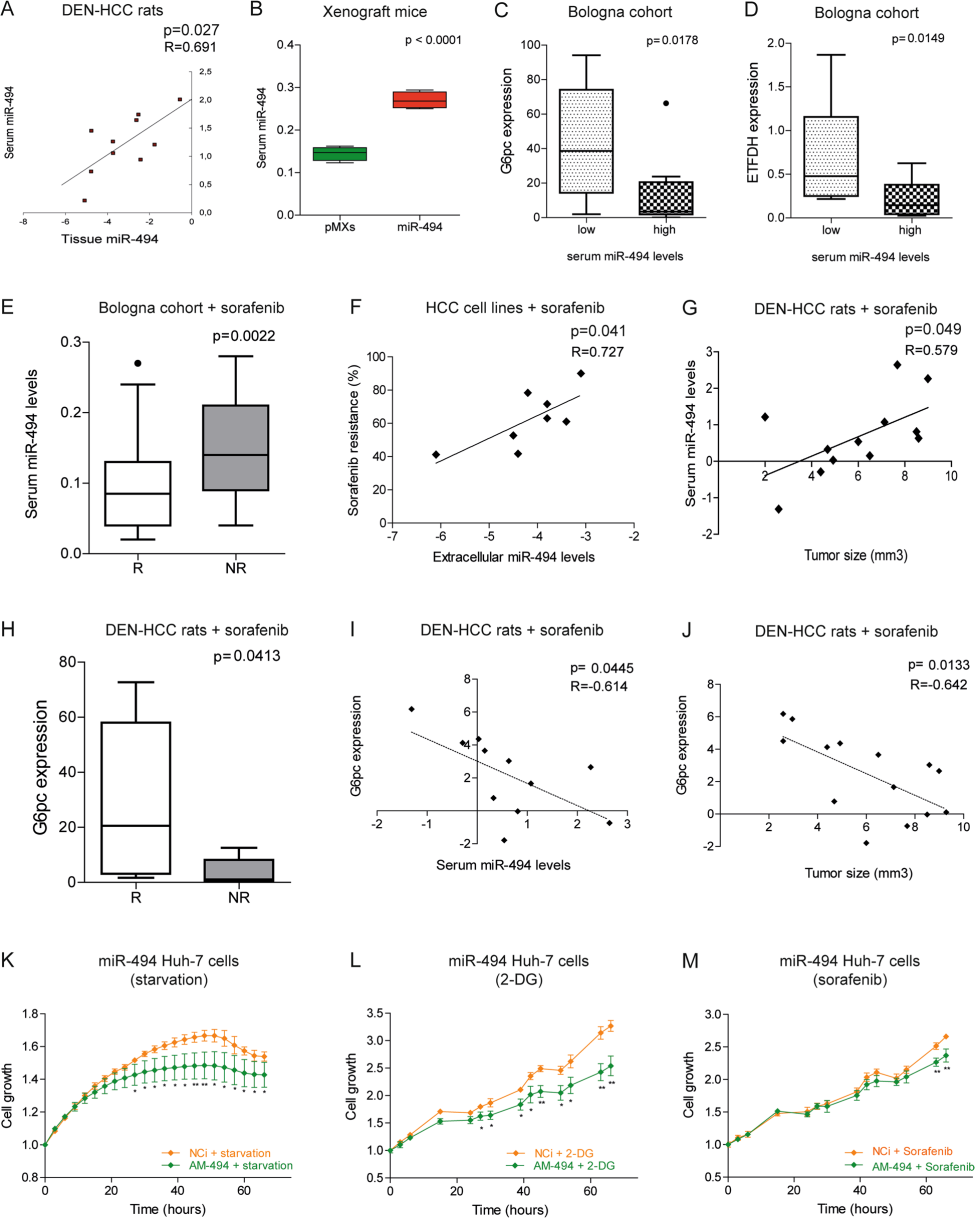
MiR-494 is a circulating biomarker and a therapeutic target in HCC. **A** Correlation graphs between tissue and serum miR-494 levels in DEN-HCC rats (N = 10). Axes report 2^−ΔΔCt^ values corresponding to tissue and serum miR-494 levels transformed in a log2 form. U6RNA and cel-miR-39 were used as control genes for tissue and circulating miRNAs, respectively. Real Time PCR analysis was run in triplicate. **B** Box plot graph of serum miR-494 levels in xenograft mice (*N* = 8) control (pMXs) and miR-494-overexpressing Huh-7 cells. Y-axis reports 2^−ΔΔCt^ values corresponding to serum miR-494 levels. Cel-miR-39 was used as control gene. Real Time PCR analysis was run in triplicate. **C** Box plot graphs of G6pc and (**D**) ETFDH tissue levels in HCC patients from the Bologna cohort expressing high (*N* = 10) and low (*N* = 12) miR-494 serum levels. Y-axes report 2^−ΔΔCt^ values corresponding to mRNA levels. GAPDH was used as housekeeping gene. Real Time PCR analysis was run in triplicate. **E** Box plot graph of baseline miR-494 serum levels in responder (R; *N* = 43) and non-responder (NR; *N* = 23) sorafenib-treated patients from the Bologna cohort. Y-axis reports 2^−ΔΔCt^ values corresponding to circulating miR-494 levels. Cel-miR-39 was used as control gene. Real Time PCR analysis was run in triplicate. **F** Correlation graph between extracellular miR-494 levels and sorafenib resistance in HCC cell lines (*N* = 8). Axes report 2^−ΔΔCt^ values corresponding to extracellular miR-494 levels transformed in a log2 form and sorafenib resistance expressed as the ratio (percent value) of cell viability between treated and untreated cells. Real Time PCR analysis was run in triplicate. **G** Correlation graph between miR-494 serum levels and tumor size in sorafenib-treated DEN-HCC rats (*N* = 12). Axes report 2^−ΔΔCt^ values corresponding to circulating miR-494 levels and tumor size (volume) of HCC nodules (mm^3^). Tumor volume was calculated with the formula V = (D1*D2*D3)/2. All the values were transformed in a log2 form. Cel-miR-39 was used as control gene. Real Time PCR analysis was run in triplicate. **H** Box plot graph of G6pc tissue levels in responder (R; *N* = 7) and non-responder (NR; *N* = 7) HCC nodules from sorafenib-treated rats. Y-axis reports 2^−ΔΔCt^ values corresponding to G6pc mRNA levels. Beta-actin was used as housekeeping gene. Real Time PCR analysis was run in triplicate. **I** Correlation graph between G6pc tissue levels and miR-494 serum levels in sorafenib-treated DEN-HCC rats (*N* = 11). Axes report 2^−ΔΔCt^ values corresponding to G6pC mRNA levels and circulating miR-494 levels transformed in a log2 form. Beta-actin and cel-miR-39 were used as control genes for tissue mRNAs and circulating miRNAs, respectively. Real Time PCR analysis was run in triplicate. **J** Correlation graph between G6pc tissue levels and tumor size of HCC nodules (*N* = 14) from sorafenib-treated DEN-HCC rats. Axes report 2^−ΔΔCt^ values corresponding to G6pC mRNA levels and tumor size (volume, mm^3^) of HCC nodules transformed in a log2 form. Beta-actin was used as housekeeping gene. Real Time PCR analysis was run in triplicate. **K** Growth curves of miR-494-overexpressing Huh-7 cells transfected with antimiR-494 (AM-494) and negative control (NCi) and cultured in serum-deprived medium or (**L**) in the presence of 2-DG or (**M**) sorafenib. Growth curves were normalized to T0. Mean ± SD values are reported. The curves were performed in two independent experiments in quadruplicate. The statistical analysis was performed using two-tailed unpaired Student's t-test and Pearson’s correlation. * *P* ≤ 0.05; ** P ≤ 0.01; *** P ≤ 0.001; **** P ≤ 0.0001

We previously demonstrated that miR-494 contributes to sorafenib resistance in HCC cell lines and showed that a combined antimiR-494-based strategy synergizes the antitumor effect of sorafenib in the DEN-HCC rat model [20]. We also reported a positive correlation between tissue and serum miR-494 levels in HCC patients and between exosome and intracellular miRNA levels in HCC cells [21]. Here, we confirmed the positive association between tissue and circulating miR-494 in two HCC animal models (Fig. 6A, B), confirming that serum miR-494 is representative of intra-tumor miRNA levels. In this regard, high miR-494 serum levels associated with lower G6pc and ETFDH expression in HCC specimens (Fig. 6C, D), letting us to hypothesize that circulating miRNA levels might be informative of liver tumors metabolism.

To investigate whether circulating miR-494 basal levels could discriminate between sorafenib responder and not-responder patients, we performed a preliminary analysis in a small case series of sorafenib-treated HCC patients. Interestingly, higher baseline miR-494 circulating levels were detected in non-responder patients from the ‘Bologna serum’ cohort (Fig. 6E). Of note, the non-responder group from the TCGA-HCC cohort displayed higher miR-494 tissue levels; despite not statistically significant due to the low number of sorafenib-treated cases (Fig. S8A). In accordance, high miR-494 extracellular levels correlated with sorafenib resistance in HCC cell lines and with increased tumor size in sorafenib-treated rats (Fig. 6F, G). Regarding G6pc, lower tissue levels associated with sorafenib resistance in rat HCCs. Moreover, G6pc expression negatively correlated with circulating miR-494 levels and tumor size, demonstrating the crucial role of miR-494/G6pc axis in sorafenib resistance in preclinical models (Fig. 6H-J).

In a treatment perspective, here we focused on miR-494-overexpressing Huh-7 cells matching the subgroup of HCC patients with high miR-494 levels and stem cell-like phenotype (25% of cases). We first investigated if antimiR-494 treatment could revert the cell growth advantage of miR-494-overexpressing cells in metabolic challenging conditions and observed that antimiR-494 transfection affected the cell growth of miRNA-overexpressing Huh-7 cells in serum-deprived medium (Fig. 6K). Subsequently, we investigated if combined strategies with antagomiR-494 could be more effective than single treatment and evaluated treatment combination with the glycolytic inhibitor 2-DG and sorafenib. Real time monitoring of the cell growth showed that antimiR-494 combination with both 2-DG and sorafenib improved treatment sensitivity (Fig. 6L, M), suggesting the therapeutic potential of miR-494 targeting when associated with metabolic interference molecules and TKIs. In line with the literature [39], we showed that the downregulation of AKT pathway is one of the molecular mechanisms involved in AM-494/sorafenib sensitization. In addition, we reported the upregulation of G6pc and the downregulation of G6PD in AM-494/sorafenib treated cells (Fig. S8B) which might confer a metabolic drawback with respect to single treatment. Finally, we performed rescue experiments in sorafenib-treated miR-494-overexpressing cells showing that G6pc overexpression restores sorafenib sensitization with respect to controls (Fig. S8C), demonstrating the influence of metabolic reprogramming of cancer cells in miR-494-mediated sorafenib resistance.

## Discussion

**Fig. 7:**
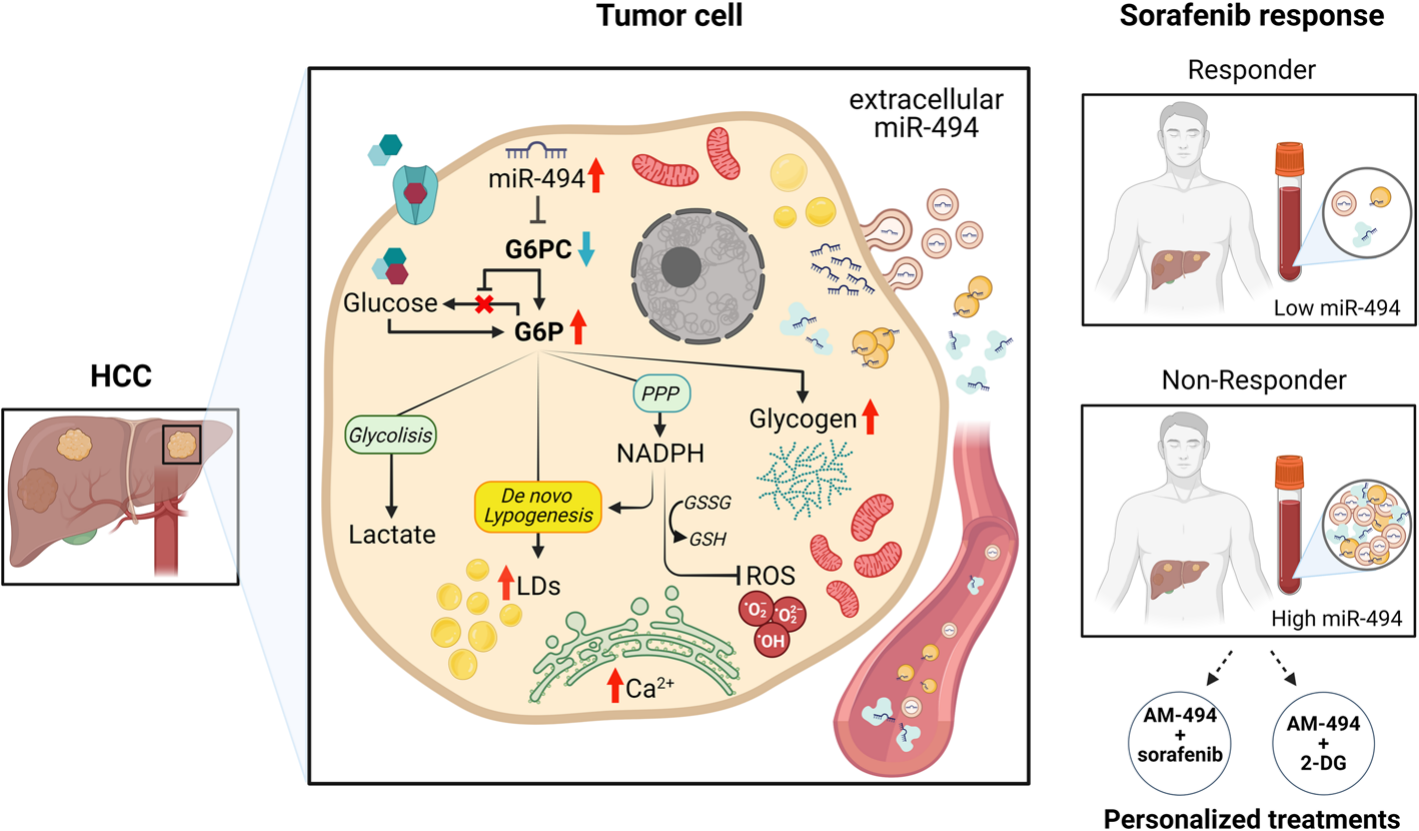
Schematic picture of miR-494/G6pc axis involvement in metabolic plasticity of HCC cells and therapeutic potential of combined antimiR-494-based strategies. Metabolic alterations occurring in tumor cells are schematized in the central part of the picture. Circulating miR-494 levels in responder and non-responder HCC patients undergoing sorafenib treatment are presented in the right part of the picture. Treatment combinations with antimiR-494 (AM-494) are proposed for non-responder patients

MiR-494 upregulation was previously identified in a subgroup of human HCCs with stem cell-like characteristics [20, 40]. By targeting MCC [41], p27, puma and pten, miR-494 was able to speed up cell cycle, enhance drug resistance, and increase invasive and clonogenic capabilities of HCC cells. Here we progressed into the characterization of miR-494 role in metabolic reprogramming of HCC cells describing how its deregulated expression affects glycolytic shift, cell survival under challenging conditions, and response to treatments interfering with cell metabolism. Aberrant miR-494 expression modified cellular metabolism by making it more plastic and resilient; in particular, it increased the glycolytic capacity of HCC cells, enabling them to cope with metabolic stress (Fig. 7). MiR-494-overexpressing cells displayed decreased respiratory control ratio, higher basal lactate production, and were less sensitive to antimycin A, indicating that they exploit anaerobic glycolysis for ATP production. Interestingly, a lactate metabolism-related gene signature associated with a shorter survival and with an immune suppressive microenvironment in HCC, highlighting its relevance not only as a prognostic biomarker but also as a predictor of treatment response [42]. In this context, an oncogene-related mouse model of HCC showed a Warburg phenotype with enhanced lactate production, PPP activation as well as impaired oxidative phosphorylation and tumor-infiltrating immune cells. The Authors reported the antitumor effect of *Ldha* knockdown inhibiting glycolytic reprogramming and restoring CD4 + lymphocytes in the tumor microenvironment [43].

Here, we identified for the first time G6pc as a miR-494 target in HCC cells, where it mediates glycolytic shift including glycogen and lipid droplets accumulation improving cell survival during nutrient deprivation. The relevance of G6pc reduction in HCC clinical course was shown by interrogating the TCGA cohort. Its reduced expression portended a worse prognosis and associated with microvascular invasion, increased tumor size and grade of HCCs. In line, other studies reported the miRNA-mediated deregulated expression of G6pc in HCC [44] and its reduced expression in different gluconeogenic tumor tissues such as clear cell renal cell carcinoma (ccRCC) [45]. G6pc is abnormally expressed in various cancer types participating to metabolic reprogramming, proliferation, invasion, and metastasis of tumor cells [46, 47]. Kim and coworker reported that GSD-Ia mice bearing less than 2% of normal hepatic G6pc activity have increased risk of HCC development, highlighting the crucial role of this gene in hepatocarcinogenesis [48]. Consistently, G6pc^−/−^ hepatocytes exhibit the main characteristics of cancer cell metabolism with Warburg-like metabolic reprogramming, decreased autophagy and antioxidant defenses, predisposing GSD-Ia livers to HCC [12, 13]. Rahim and coworkers reported that G6pc2 knockout βTC3 cells displayed a significant elevation in cytoplasmic calcium levels [49]; this observation fits well with the higher free calcium levels detected in miR-494-overexpressing cells. Of note, Liang and coworkers reported calcium signaling and cell metabolism as the most frequently deregulated/mutated pathways in high-fat/high-cholesterol NASH-HCC mice and human NASH-HCCs, pinpointing their importance for inflammatory-based NAFLD progression and opening the path for the identification of new therapeutic strategies [50]. Moreover, calcium signaling pathways interact with other cellular signaling systems, including ROS [51]. When in excess, ROS lead to molecular damage and cause cell distress, especially in highly proliferative tumor cells that, in turn, activate signaling pathways able to counteract their production. MiR-494-overexpressing cells displayed enhanced glutathione levels, making them able to cope with increased intracellular and mitochondrial ROS levels and finally resulting in diminished lipid peroxidation. During glucose deprivation, miR-494-overexpressing cells exhibited lower oxidative stress and increased glycogen breakdown. Because glycogen demolition leads to the production of G6P, a key metabolite in the PPP that contributes to the reconversion of oxidized glutathione into its reduced form, we hypothesized that the ability of miR-494-overexpressing cells to counterbalance oxidative stress under metabolic adverse conditions might be related to their ability to accumulate and demolish glycogen when needed. In agreement, a recent study demonstrated G6pc-mediated glycogen accumulation as a driver event for liver cancer development via Hippo pathway switching off [52].

We previously reported the early downregulation of the metabolic gene HAO2 in human and rodent HCCs concurring to lowering lipid peroxidation and oxidative stress in tumor cells [38]. Xiao et al. found decreased HAO2 levels in ccRCC and demonstrated that its overexpression inhibits the malignancy of cancer cells by eliminating lipid droplet accumulation and promoting intracellular lipid catabolism [53]. In this context, we can speculate that the downregulation of HAO2 mRNA in miR-494-ovexpressing cells might contribute not only to lowering down oxidative stress, but also to accumulate lipid droplets sustaining increased proliferation and metabolic plasticity in harsh environmental conditions.

Alteration of lipid metabolism represents a hallmark of cancer and lipid remodeling is present in proliferating hepatocytes and HCC [54]. Lipid reprogramming is mainly characterized by increased de novo fatty acid (FA) and cholesterol synthesis and impaired FA β-oxidation. An elegant study reported the relationship between mitochondrial fission and lipid reprogramming in preclinical models of HCC inducing proliferation and metastasis of tumor cells [55]. The same Authors also demonstrated the induction of carbohydrate reprogramming towards OXPHOS by mitochondrial fission suppression in cancer cells [56]. In general, oxidative metabolism is less active in fragmented mitochondria than in highly interconnected mitochondria because limiting OXPHOS preserves glycolytic intermediates, which can be used for cancer cell proliferation. On the other hand, a highly activated glycolysis has been linked to mitochondrial fission in many cancer types when high respiratory activity is not required [57]. Similarly, we observed impaired mitochondrion morphology in miR-494-overexpressing cells, together with gene expression activation of key enzymes involved in FA and triglyceride synthesis (e.g.: FASN and LPIN1) leading to a prolonged survival under glucose-free conditions. Lipin-1 is known to regulate the lipid metabolism during fasting adaptation [58] and appears critical for the survival of cancer cells [59]; besides, it catalyzes a key step in the biosynthesis of triacylglycerol and phospholipids, which are essential for highly replicative cells. PGC-1alpha downregulation observed in miR-494-overexpressing cells might contribute to deregulate further G6pc transcription and to inhibit lipid catabolism, highlighting the multi-targeting nature of miR-494 and its deep impact on lipid metabolism in HCC. Rescue experiments with G6pc overexpression vector in miR-494-overexpressing cells lowered lipid content and cell survival in harsh metabolic conditions, confirming its critical role in miR-494-based metabolic reprogramming of HCC. Notably, several studies investigated FASN inhibitors as promising alternative strategies to hit altered lipid synthesis, but their toxicity profiles prevented their introduction into the clinics. On the other hand, promising preclinical studies with well-tolerated lipogenesis inhibitors are ongoing in other malignancies [60]. In this scenario, an antagomiR-494-based strategy might be an appealing therapeutic option in the subgroup of patients with high miRNA levels and stem cell-like features. Indeed, it seems conceivable that miR-494, being a direct modulator of G6pc, as well as of other genes including HIF1A, might represent a putative therapeutic target to be exploited in future research.

We also evaluated whether miR-494 inhibition might enhance the effect of current HCC treatments. We previously demonstrated that miR-494 contributes to sorafenib resistance via mTOR pathway activation in HCC cells and showed that miR-494 inhibition enhances the anti-tumor effect of sorafenib in rats [20]. Despite the combination of atezolizumab with bevacizumab is currently the first choice first-line treatment [61], not all patients can benefit from immunotherapy and, in the latter cases, sorafenib and lenvatinb represent the first line treatments of choice [62]. Here, we confirmed the synergic effect of antimiR-494/sorafenib treatment and demonstrated for the first time that, together with AKT pathway repression, G6pc targeting mediates miR-494-induced sorafenib resistance in HCC cells. In line, the oncomiR-21 triggered sorafenib resistance in HCC cells by PTEN direct targeting or by regulating the nuclear localization of the long non-coding RNA SNHG1 [63]. The downregulation of G6PD, the key and rate-limiting enzyme in the PPP, might also explain the antimiR-494-mediated sorafenib sensitization through an impaired control of ROS production. Similarly, Wei and coworkers demonstrated a synergistic anticancer activity of phosphoglycerate dehydrogenase (PHGDH) blocking in combination with sorafenib treatment, leading to reduced α-ketoglutarate, serine, and NADPH levels in the presence of increased ROS production [64], suggesting the alteration of the oxidative potential of cancer cells as a further mechanism through which miR-494 inhibition might favor sorafenib sensitivity in HCC. In agreement, miR-23a-3p influenced sorafenib response by blocking ferroptosis leading to reduced iron accumulation and lipid peroxidation in HCC preclinical models [65]. In summary, here we show that antagomiR-494/sorafenib combination deserves attention for the treatment of a well-defined HCC patient subgroup. Nevertheless, we think that before testing this combination in clinical trials, it should be tested in other HCC animal models also evaluating different miRNA-delivery strategies.

Finally, we performed explorative analysis to investigate if miR-494 could represent a possible non-invasive biomarker for the identification of metabolic-related HCCs and for the evaluation of sorafenib response. On one hand, high miR-494 circulating levels associated with low tissue metabolic gene (G6pc and ETFDH) expression, highlighting the potential of identifying an HCC subgroup with miR-494-related metabolic alterations that might represent a treatable vulnerability to complement actual therapies. On the other hand, higher miR-494 serum levels associated with sorafenib resistance in advanced patients. Despite these encouraging findings, we are aware that, to evaluate the predictive power of miR-494 as a serum biomarker, larger validation prospective case series of HCC patients would be needed.

In the light of miR-494 role in metabolic reprogramming, we evaluated the therapeutic potential of combined strategies in HCC cells. We showed that the positive effect of miR-494 inhibition on sorafenib sensitization of Huh-7 cells is mediated, at least in part, by G6pc targeting. Notably, we also reported a synergistic effect when combining antimiR-494 with the glycolysis interference molecule 2-DG. Based on promising preclinical studies and clinical trials with metabolic inhibitors [66] and considering the influence of cancer cell metabolism on the fitness of infiltrating immune cells [67], as well as the immune tolerance of metabolic HCCs [68], our findings pave the way toward novel miRNA/metabolic-based therapeutic perspectives in HCC.

## Conclusions

In summary, we reported that miR-494/G6pc axis, together with HIF-1A activation, triggers metabolic reprogramming of HCC cells toward a glycolytic phenotype, regulating glycogen and lipids storage to deal with challenging metabolic conditions. Here, we provided new molecular insights for the identification of treatment combinations for well-defined patient subgroups with reduced susceptibility to actual therapies. Finally, we have shown that high circulating levels of miR-494 associate with sorafenib resistance in a preliminary cohort of HCC patients and in preclinical models, suggesting future investigations of miR-494 as a possible biomarker for treatment personalization in HCC.

## Supplementary Information

**Additional file 1.** Supplementary Tables.

**Additional file 2.** Supplementary Figures.

**Additional file 3.** Bioinformatics analysis.

## Data Availability

Raw data from the microarray analysis of DEN-HCC rat tissues are available in ArrayExpress repository database (accession number E-MTAB-11977) at EMBL-EBI (www.ebi.ac.uk/arrayexpress).

## Abbreviations

HCC: Hepatocellular Carcinoma
G6pc: Glucose 6-phosphatase catalytic subunit
NAFLD: Nonalcoholic fatty liver disease
NASH: Nonalcoholic steatohepatitis
G6P: Glucose-6 phosphate
PPP: Pentose phosphate pathway
GSD-Ia: Glycogen storage disease type Ia
OXPHOS: Oxidative phosphorylation
MicroRNA: MiR
mTOR: Mammalian target of rapamycin
TCGA: The Cancer Genome Atlas
DEN: Diethylnitrosamine
PAS: Periodic Acid Schiff
WB: Western blot
HIF1A: Hypoxia-inducible factor
GO: Gene ontology
CC: Cellular component
BP: Biologic processes
TKI: Tyrosine Kinase Inhibitors
3’UTR: 3’-Untranslated region
OCR: Oxygen consumption rate
SDH: Succinate dehydrogenase
CS: Citrate synthase
LDH: Lactate dehydrogenase
AA: Antimycin A
DEA: Data envelopment analysis
LDs: Lipid droplets
2-DG: 2-Deoxy-glucose
ROS: Reactive oxygen species
HAO2: Hydroxy acid oxidase 2
ccRCC: Clear cell renal cell carcinoma
