# Supplementary material for: MiR-494 induces metabolic changes through G6pc targeting and modulates sorafenib response in hepatocellular carcinoma
Source: J Exp Clin Cancer Res. 2023 Jun 10;42:145. doi: 10.1186/s13046-023-02718-w (PMC10257313; doi:10.1186/s13046-023-02718-w)
Supplement: Supplementary file 1 — Additional file 1. Supplementary Tables. [file 13046_2023_2718_MOESM1_ESM.docx]

# Supplementary Table 1 – Characteristics of the surgical cohort (N=46) of HCC patients (Bologna cohort)

| **Clinical Factors** |  | **Frequency (%)**  **46 pts** |
| --- | --- | --- |
| Gender | Male/Female | 34/12 |
| Age | <65 yrs | 9/46 (19.6%) |
| Etiology | HBV | 7/46 (15.2%) |
|  | HCV | 31/46 (67.4%) |
|  | Alcohol abuse | 2/46 (4.3%) |
|  | NASH/NAFLD | 5/46 (10.9%) |
|  | None | 1/46 (2.2%) |
| Cirrhosis |  | 42/46 (91.3%) |
| Serum AFP | > 20 ng/ml | 29/46 (63%) |
| Edmondson grade | I | 0% |
|  | II | 9/46 (19.6%) |
|  | III | 31/46 (67.4%) |
|  | IV | 6/46 (13%) |
| Barcelona Clinic Liver Cancer stage (BCLC) | A | 35/46 (76.1%) |
|  | B | 11/46 (23.9%) |
|  | C | 0% |
|  | D | 0% |

# Supplementary Table 2 – Characteristics of the cohort of advanced HCC patients (N=65) subjected to sorafenib treatment (Bologna cohort serum)

|  |  | **Responders**  **(N=43)** | **Non responders**  **(N=23)** | **Difference** |
| --- | --- | --- | --- | --- |
| Age | <65 yrs | 12 (27.92%) | 8 (34.8%) |  |
|  | >65 yrs | 31 (72.1%) | 15 (65.2%) | n.s. |
| Gender | Male | 33 (76.7%) | 15 (65.2%) |  |
|  | Female | 10 (23.3%) | 8 (34.8%) | n.s. |
| Viral infection | HBV | 4 (9.3%) | 4 (17.4%) |  |
|  | HCV | 31 (72.1%) | 14 (60.9%) |  |
|  | NASH/NAFLD | 8 (18.6%) | 5 (21.7%) | n.s. |
| Stage of HCC | intermediate | 5 (11.6%) | 3 (13%) |  |
|  | advanced | 38 (88.4%) | 20 (86.9%) | n.s. |
| AFP (ng/mL) | <20 | 19 (44.2%) | 12 (52.2%) |  |
|  | >20 | 24 (55.8%) | 11 (47.8%) | n.s. |
| Child-Pugh | A5 | 36 (83.7%) | 17 (73.9%) |  |
|  | A6 | 7 (16.3%) | 6 (26.1%) | n.s. |
| Portal vein invasion | Yes | 6 (14.0%) | 8 (34.8%) |  |
|  | No | 37 (86.0%) | 15 (65.2%) | n.s. |
| Circulating miR-494 | High | 18 (41.9%) | 16 (69.6%) |  |
|  | Low | 25 (58,1%) | 7 (30.4%) | 0.032 |

# Supplementary Table 3 – Primer sequences for Real Time PCR and HIF-1A DsiRNAs sequence

| Gene | Primer sequence |
| --- | --- |
| G6pc | Fw 5’-AGGGAAAGATAAAGCCGACC-3’  Rv 5’-AGCAAGGTAGATTCGTGACAG-3’ |
| ß-ACTIN | Fw 5’-ACCTTCTACAATGAGCTGCG-3’  Rv 5’-CCTGGATAGCAACGTACATGG-3’ |
| GAPDH | Fw 5’-ACATCGCTCAGACACCATG-3’  Rv 5’-TGTAGTTGAGGTCAATGAAGGG-3’ |
| LPIN1 | Fw 5’-GTCAGCCTCATACCCTAATTCG-3’  Rv 5’-CTTCTGCCCTGTCCTTTCC-3’ |
| FASN | Fw 5’-CAAGCTGAAGGACCTGTCTAG-3’  Rv 5’-CGGAGTGAATCTGGGTTGATG-3’ |
| ETFDH | Fw 5’-TTCAACTTCTACTGTGCCTCG-3’  Rv 5’-GCCTGCACCAACTATTACAAC-3’ |
| G6PD | Fw 5’-AGAACATTCACGAGTCCTGC-3’  Rv 5’-GTGGTCGATGCGGTAGATC-3’ |
| CS | Fw 5’-CATTGACTCTAACCTGGACTGG-3’  Rv 5’-ACTTACATTGCCACCCTCATG-3’ |
| SDH | Fw 5’-TGGTTGTCTTTGGTCGGG-3’  Rv 5’-GCGTTTGGTTTAATTGGAGGG-3’ |
| PGC1A | Fw 5’-ACCAAACCCACAGAGAACAG-3’  Rv 5’-GGGTCAGAGGAAGAGATAAAGTTG-3’ |
| HAO2 | Fw 5’-GCTGTCTAAGTCAACTCGGG-3’  Rv 5’-TGGATTGTGGTTCTGGTGTC-3’ |
| HIF1A | Fw 5’-AACATAAAGTCTGCAACATGGAAG-3’  Rv 5’-TTTGATGGGTGAGGAATGGG-3’ |
| ALDOA | Fw 5’-GGTGTCATCCTCTTCCATGAG-3’  Rv 5’-GTAGTCTCGCCATTTGTCCC-3’ |
| GLUT1 | Fw 5’-AAAGTGACAAGACACCCGAG-3’  Rv 5’-TGTCAGGTTTGGAAGTCTCATC-3’ |
| Rat_G6PC | Fw 5’-GTCTTGTGGTTGGGATACTGG-3’  Rv 5’-CGGATGTGGCTGAAAGTTTC-3’ |
| Rat_ß-ACTIN | Fw 5’-CACTTTCTACAATGAGCTGCG-3’  Rv 5’-CTGGATGGCTACGTACATGG-3’ |
| HIF1A.13.1 DsiRNA A | Fw 5’-UCACCAAAGUUGAAUCAGAAGAUAC-3’  Rv 5’-GUAUCUUCUGAUUCAAUUUGGUGAAU-3’ |
| HIF1A.13.2 DsiRNA B | Fw 5’CUGAUGUUUCUAUAGUCACUUUGCC-3’  Rv 5’-GGCAAAGUGACUAUAGAAACAUCAGAU-3’ |
| HIF1A.13.3 DsiRNA C | Fw 5’-CGGUUGAAUCUUCAGAUAUGAAAAT-3’  Rv 5’-AUUUUCAUAUCUGAAGAUUAACCGGU-3’ |

# Supplementary Table 4 - Antibodies for WB analysis

| Antibody | Catalogue number | Company |
| --- | --- | --- |
| G6PC | #PA5-42541 | Invitrogen |
| HIF-1 | #E-AB-31662 | ElabScience |
| β-actin (C4) | #sc-47778 | Santa Cruz Biotechnology |
| G6PD (G-12) | #sc-373886 | Santa Cruz Biotechnology |
| AKT | #9272 | Cell Signaling Technologies |
| phospo-AKT (Ser473) (D9E) XP | #4060 | Cell Signaling Technologies |
| Cleaved caspase-3 (Asp175) | #9661 | Cell Signaling Technologies |
| PARP | #9542 | Cell Signaling Technologies |

# Supplementary Table 5 – Primer sequences for cloning and mutagenesis analysis

| Cloned region | Primer sequence | Amplified product (bp) | Annealing T (°C) |
| --- | --- | --- | --- |
| G6PC-3’UTR | Fw 5’-CCATG**TCTAGA**TGGCTTTAATTATATAG-3’  Rv 5’-GCCCAGGAAT**TCTAGA**GCAG-3’ | 843 | 60 |
| G6PC-mut | Fw 5’-CCCAAGATTTCAGAGCCTAATTG**CCC**CATACAAAAGCACCACCAGAGTC-3’  Rv 5’-GACTCTGGTGGTGCTTTTGTATG**GGG**CAATTAGGCTCTGAAATCTTGGG-3’ | - | 78 |

# Supplementary Table 6 – MiR-494 hypothetic target genes downregulated in HCC

| GENE SYMBOL | FULL NAME |
| --- | --- |
| *BBOX1* | butyrobetaine (gamma), 2-oxoglutarate dioxygenase (gamma-butyrobetaine hydroxylase) 1 |
| *XKR4* | XK related 4 |
| *PROS1* | protein S |
| *ACBD4* | acyl-CoA binding domain containing 4 |
| *DPYD* | dihydropyrimidine dehydrogenase |
| *FCN1* | ficolin (collagen/fibrinogen domain containing) 1 |
| *LRAT* | lecithin retinol acyltransferase (phosphatidylcholine--retinol O-acyltransferase) |
| *CYB5A* | cytochrome b5 type A |
| *SLC25A47* | solute carrier family 25, member 47 |
| *HADH* | hydroxyacyl-CoA dehydrogenase |
| *GRAMD1C* | GRAM domain containing 1C |
| *BCHE* | butyrylcholinesterase |
| *TIMD4* | T-cell immunoglobulin and mucin domain containing 4 |
| *TCAIM* | T cell activation inhibitor, mitochondrial |
| *PNPLA7* | patatin-like phospholipase domain containing 7 |
| *CD274* | CD274 molecule |
| *SOCS2* | suppressor of cytokine signaling 2 |
| *IL1R1* | interleukin 1 receptor type 1 |
| *PELI2* | pellino E3 ubiquitin protein ligase family member 2 |
| *LCAT* | lecithin-cholesterol acyltransferase |
| *RCAN1* | regulator of calcineurin 1 |
| *TGFBR3* | transforming growth factor, beta receptor III |
| *TTPA* | alpha tocopherol transfer protein |
| *FMO2* | flavin containing monooxygenase 2 (non-functional) |
| *PANK1* | pantothenate kinase 1 |
| *CD5L* | CD5 molecule-like |
| *TDO2* | tryptophan 2,3-dioxygenase |
| *ACVR1C* | activin A receptor type 1C |
| *BCKDHB* | branched chain keto acid dehydrogenase E1, beta polypeptide |
| *SLC7A2* | solute carrier family 7 (cationic amino acid transporter, y+ system), member 2 |
| *IDO2* | indoleamine 2,3-dioxygenase 2 |
| *NMRK1* | nicotinamide riboside kinase 1 |
| *ST6GAL1* | ST6 beta-galactoside alpha-2,6-sialyltransferase 1 |
| *CLEC1B* | C-type lectin domain family 1, member B |
| *HAND2* | heart and neural crest derivatives expressed 2 |
| *DMGDH* | dimethylglycine dehydrogenase |
| *RMDN2* | regulator of microtubule dynamics 2 |
| *LRRC4C* | leucine rich repeat containing 4C |
| *NAALADL2* | N-acetylated alpha-linked acidic dipeptidase-like 2 |
| *SLC25A25* | solute carrier family 25 (mitochondrial carrier; phosphate carrier), member 25 |
| *CXCL12* | chemokine (C-X-C motif) ligand 12 |
| *S1PR1* | sphingosine-1-phosphate receptor 1 |
| *G6PC* | glucose-6-phosphatase, catalytic subunit |
| *CELF2* | CUGBP, Elav-like family member 2 |
| *INSIG2* | insulin induced gene 2 |
| *FABP1* | fatty acid binding protein 1, liver |
| *BCO2* | beta-carotene oxygenase 2 |
| *F9* | coagulation factor IX |
| *SULT2A1* | sulfotransferase family, cytosolic, 2A, dehydroepiandrosterone (DHEA)-preferring, member 1 |
| *RNF144B* | ring finger protein 144B |
| *AGMO* | alkylglycerol monooxygenase |
| *NFIA* | nuclear factor I A |
| *GPR146* | G protein-coupled receptor 146 |
| *IL18R1* | interleukin 18 receptor 1 |
| *SRD5A1* | steroid-5-alpha-reductase, alpha polypeptide 1 (3-oxo-5 alpha-steroid delta 4-dehydrogenase alpha 1) |
| *NRXN1* | neurexin 1 |
| *FAM126B* | hyccin PI4KA lipid kinase complex subunit 2 |
| *CMYA5* | cardiomyopathy associated 5 |
| *TTC39B* | tetratricopeptide repeat domain 39B |
| *IL33* | interleukin 33 |
| *SLC25A16* | solute carrier family 25 member 16 |
| *SC5D* | sterol-C5-desaturase |
| *GHR* | growth hormone receptor |
| *ENPEP* | glutamyl aminopeptidase |
| *PDGFC* | platelet derived growth factor C |
| *CP* | ceruloplasmin (ferroxidase) |
| *ADHFE1* | alcohol dehydrogenase iron containing 1 |
| *AR* | androgen receptor |
| *SLC25A13* | solute carrier family 25 member 13 |
| *ACACB* | acetyl-CoA carboxylase beta |
| *TAPT1* | transmembrane anterior posterior transformation 1 |
| *MBL2* | mannose-binding lectin (protein C) 2, soluble |
| *MCC* | mutated in colorectal cancers |
| *RAPGEF4* | Rap guanine nucleotide exchange factor 4 |
| *PDK4* | pyruvate dehydrogenase kinase, isozyme 4 |
| *NIPAL1* | NIPA-like domain containing 1 |
| *SLC16A10* | solute carrier family 16 member 10 |
| *NEGR1* | neuronal growth regulator 1 |
| *AVPR1A* | arginine vasopressin receptor 1A |
| *TAT* | tyrosine aminotransferase |
| *IGF1* | insulin-like growth factor 1 (somatomedin C) |
| *RCL1* | RNA terminal phosphate cyclase-like 1 |
| *KMO* | kynurenine 3-monooxygenase (kynurenine 3-hydroxylase) |
| *CALN1* | calneuron 1 |
| *CBS* | cystathionine-beta-synthase |
| *SEMA3D* | sema domain, immunoglobulin domain (Ig), short basic domain, secreted, (semaphorin) 3D |
| *PPP1R3B* | protein phosphatase 1, regulatory subunit 3B |
| *ABHD15* | abhydrolase domain containing 15 |
| *FAHD2A* | fumarylacetoacetate hydrolase domain containing 2A |
| *C1S* | complement component 1, s subcomponent |
| *ZFYVE21* | zinc finger FYVE-type containing 21 |
| *GNE* | glucosamine (UDP-N-acetyl)-2-epimerase/N-acetylmannosamine kinase |
| *NADK2* | NAD kinase 2, mitochondrial |
| *PIK3R1* | phosphoinositide-3-kinase regulatory subunit 1 |
| *CLDN1* | claudin 1 |
| *CTH* | cystathionase (cystathionine gamma-lyase) |
| *ZADH2* | zinc binding alcohol dehydrogenase domain containing 2 |
| *ANTXR2* | anthrax toxin receptor 2 |
| *STEAP4* | STEAP family member 4 |
| *SORL1* | sortilin-related receptor, L(DLR class) A repeats containing |
| *DIXDC1* | DIX domain containing 1 |
| *SNED1* | sushi, nidogen and EGF-like domains 1 |
| *APOA5* | apolipoprotein A-V |
| *ABAT* | 4-aminobutyrate aminotransferase |
| *KYNU* | kynureninase |
| *ACSM3* | acyl-CoA synthetase medium-chain family member 3 |
| *RNF125* | ring finger protein 125, E3 ubiquitin protein ligase |
| *SLC6A12* | solute carrier family 6 (neurotransmitter transporter), member 12 |
| *ACADM* | acyl-CoA dehydrogenase medium chain |
| *HGF* | hepatocyte growth factor (hepapoietin A; scatter factor) |
| *LCA5* | Leber congenital amaurosis 5 |
| *PTPRB* | protein tyrosine phosphatase, receptor type, B |
| *EXPH5* | exophilin 5 |
| *KLF9* | KLF transcription factor 9 |
| *LIPC* | lipase, hepatic |
| *CREM* | cAMP responsive element modulator |
| *AGXT* | alanine-glyoxylate aminotransferase |
| *TCF21* | transcription factor 21 |
| *SFXN2* | sideroflexin 2 |
| *TSKU* | tsukushi, small leucine rich proteoglycan |
| *PPM1K* | protein phosphatase, Mg2+/Mn2+ dependent, 1K |
| *BDH1* | 3-hydroxybutyrate dehydrogenase, type 1 |
| *SIRT3* | sirtuin 3 |
| *PAH* | phenylalanine hydroxylase |
| *LONP2* | lon peptidase 2, peroxisomal |
| *ST3GAL6* | ST3 beta-galactoside alpha-2,3-sialyltransferase 6 |
| *GRHPR* | glyoxylate reductase/hydroxypyruvate reductase |
| *ECHDC3* | enoyl-CoA hydratase domain containing 3 |
| *CMBL* | carboxymethylenebutenolidase homolog |
| *PQLC1* | solute carrier family 66 member 2 |
| *GLYAT* | glycine-N-acyltransferase |
| *DUSP1* | dual specificity phosphatase 1 |
| *ATOH8* | atonal homolog 8 (Drosophila) |
| *ANG* | angiogenin, ribonuclease, RNase A family, 5 |
| *VNN3* | vanin 3 |
| *CD163* | CD163 molecule |
| *RSPO3* | R-spondin 3 |
| *ANGPTL3* | angiopoietin-like 3 |
| *ALDH2* | aldehyde dehydrogenase 2 family (mitochondrial) |
| *OTC* | ornithine carbamoyltransferase |
| *GDF2* | growth differentiation factor 2 |
| *PLG* | plasminogen |
| *EHD3* | EH-domain containing 3 |
| *COLEC10* | collectin sub-family member 10 (C-type lectin) |

# Supplementary Table 7 – Metabolic pathways gene list

| Gene Symbol | DEN-HCC rat model | | TCGA human HCC | |
| --- | --- | --- | --- | --- |
|  | **LogFC** | **p-value** | **LogFC** | **p-value** |
| SULT2A1 | -3.15331 | 6.86E-05 | -1.20327 | 2.41E-05 |
| DMGDH | -2.85935 | 3.07E-05 | -1.43234 | 8.18E-11 |
| ADHFE1 | -2.66947 | 0.000109 | -0.83004 | 4.63E-06 |
| G6PC | **-2.63742** | **0.000169** | **-1.11065** | **0.000161** |
| CBS | -2.61798 | 0.000144 | -1.0171 | 6.82E-06 |
| AGXT | -2.49219 | 0.000186 | -1.05521 | 9.14E-05 |
| TDO2 | -2.48245 | 0.000258 | -1.72667 | 4.2E-08 |
| LRAT | -2.4442 | 0.001294 | -2.96145 | 9.43E-24 |
| GLYAT | -2.38765 | 0.000328 | -1.96487 | 8.91E-09 |
| DPYD | -2.2499 | 4.85E-05 | -0.85455 | 6.26E-06 |
| CTH | -2.22653 | 0.00014 | -1.33937 | 2.49E-07 |
| BBOX1 | -2.21123 | 0.000627 | -2.05969 | 2.89E-09 |
| APOA5 | -2.19143 | 0.000896 | -1.04375 | 0.000177 |
| AGMO | -2.13881 | 9.75E-05 | -0.7623 | 0.001065 |
| BCO2 | -2.00851 | 3.89E-05 | -3.34881 | 1.06E-26 |
| FMO2 | -2.00829 | 0.000128 | -1.33103 | 9.83E-07 |
| IDO2 | -1.99738 | 0.0001 | -2.28361 | 1.9E-07 |
| INSIG2 | -1.99498 | 6.63E-05 | -0.44255 | 0.006077 |
| PPM1K | -1.9175 | 4.99E-05 | -0.6721 | 0.00472 |
| PDK4 | -1.89497 | 2.82E-05 | -1.2799 | 9.6E-07 |
| KYNU | -1.79048 | 0.002669 | -0.72312 | 0.00345 |
| ACADM | -1.76363 | 1.67E-05 | -0.92252 | 1.82E-08 |
| PAH | -1.73936 | 0.000746 | -0.94541 | 9.03E-06 |
| SRD5A1 | -1.72028 | 0.000246 | -1.14017 | 1.5E-09 |
| RAPGEF4 | -1.62614 | 2.46E-05 | -0.59596 | 0.00045 |
| FABP1 | -1.60604 | 0.000137 | -1.40484 | 6.84E-05 |
| GRHPR | -1.54725 | 0.000123 | -1.11513 | 2.02E-11 |
| LIPC | -1.5426 | 6.98E-05 | -1.1593 | 3.33E-06 |
| TAT | -1.51819 | 0.000637 | -1.10394 | 0.0016 |
| LCAT | -1.51782 | 2.16E-06 | -2.70598 | 3.93E-24 |
| BDH1 | -1.44954 | 0.000191 | -0.9272 | 6.59E-10 |
| SLC6A12 | -1.43598 | 0.000244 | -0.88184 | 0.000795 |
| CYB5A | -1.43155 | 0.000282 | -0.9327 | 1.33E-09 |
| SLC25A13 | -1.41416 | 5.86E-06 | -0.55638 | 3.83E-05 |
| PIK3R1 | -1.39166 | 1.86E-06 | -0.64214 | 0.000961 |
| KMO | -1.3743 | 0.000308 | -1.49078 | 6.26E-07 |
| HADH | -1.2452 | 4.87E-06 | -0.6997 | 3.94E-06 |
| ACACB | -1.2407 | 0.000424 | -1.14148 | 7.52E-11 |
| BCKDHB | -1.16928 | 0.000232 | -1.19791 | 9.49E-12 |
| OTC | -1.16055 | 0.0085 | -0.99106 | 0.000262 |
| PANK1 | -1.15763 | 0.000172 | -1.4658 | 1.19E-18 |
| CMBL | -1.0943 | 0.040586 | -0.98197 | 1.6E-05 |
| ST3GAL6 | -1.07647 | 0.000106 | -1.36007 | 1.66E-12 |
| SC5D | -1.06948 | 8.75E-07 | -0.63283 | 0.002044 |
| SLC25A16 | -1.06818 | 1.31E-05 | -0.53273 | 2.39E-07 |
| ALDH2 | -1.04732 | 1.89E-05 | -1.46058 | 4.74E-19 |
| PNPLA7 | -1.01931 | 0.000268 | -1.51418 | 5.2E-13 |
| BCHE | -1.00148 | 0.003861 | -1.95941 | 3.63E-08 |

# Supplementary Table 8 - Downregulated mitochondrial and lipid metabolism-associated genes in miR-494 high expressing TCGA HCC tumors

| **Symbol** | **Mitochondrial localization** | **Mitochondrial Pathway** |
| --- | --- | --- |
| NDUFS7 | MIM | OXPHOS > Complex I > CI subunits \| Metabolism > Metals and cofactors > Fe-S-containing proteins \| OXPHOS > OXPHOS subunits |
| NDUFS2 | MIM | OXPHOS > Complex I > CI subunits \| Metabolism > Metals and cofactors > Fe-S-containing proteins \| OXPHOS > OXPHOS subunits |
| NDUFAF1 | MIM | OXPHOS > Complex I > CI assembly factors \| OXPHOS > OXPHOS assembly factors |
| NDUFA7 | MIM | OXPHOS > Complex I > CI subunits \| OXPHOS > OXPHOS subunits |
| NDUFA8 | MIM | OXPHOS > Complex I > CI subunits \| OXPHOS > OXPHOS subunits |
| NDUFA1 | MIM | OXPHOS > Complex I > CI subunits \| OXPHOS > OXPHOS subunits |
| ECSIT | MIM | OXPHOS > Complex I > CI assembly factors \| OXPHOS > OXPHOS assembly factors |
| TMEM70 | MIM | OXPHOS > Complex I > CI assembly factors \| OXPHOS > Complex V > CV assembly factors \| OXPHOS > OXPHOS assembly factors |
| NDUFC1 | MIM | OXPHOS > Complex I > CI subunits \| OXPHOS > OXPHOS subunits |
| AIFM1 | MIM | Protein import, sorting and homeostasis > Protein import and sorting > MIA40 \| OXPHOS > Complex I > CI assembly factors \| OXPHOS > OXPHOS assembly factors \| Mitochondrial dynamics and surveillance > Apoptosis |
| NDUFS4 | MIM | OXPHOS > Complex I > CI subunits \| OXPHOS > OXPHOS subunits |
| SDHD | MIM | OXPHOS > Complex II > CII subunits \| Metabolism > Carbohydrate metabolism > TCA cycle \| Metabolism > Metals and cofactors > Heme-containing proteins \| OXPHOS > OXPHOS subunits |
| SDHC | MIM | OXPHOS > Complex II > CII subunits \| Metabolism > Carbohydrate metabolism > TCA cycle \| Metabolism > Metals and cofactors > Heme-containing proteins \| OXPHOS > OXPHOS subunits |
| UQCRC1 | MIM | Protein import, sorting and homeostasis > Protein import and sorting > Preprotein cleavage \| OXPHOS > Complex III > CIII subunits \| OXPHOS > OXPHOS subunits |
| UQCRQ | MIM | OXPHOS > Complex III > CIII subunits \| OXPHOS > OXPHOS subunits |
| UQCR10 | MIM | OXPHOS > Complex III > CIII subunits \| OXPHOS > OXPHOS subunits |
| NDUFA4 | MIM | OXPHOS > Complex IV > CIV subunits \| OXPHOS > OXPHOS subunits |
| COA3 | MIM | Mitochondrial central dogma > Translation > Translation factors \| OXPHOS > Complex IV > CIV assembly factors \| OXPHOS > OXPHOS assembly factors |
| COX4I2 | MIM | OXPHOS > Complex IV > CIV subunits \| OXPHOS > OXPHOS subunits |
| COX18 | MIM | OXPHOS > Complex IV > CIV assembly factors \| OXPHOS > OXPHOS assembly factors |
| COX6A1 | MIM | OXPHOS > Complex IV > CIV subunits \| OXPHOS > OXPHOS subunits |
| COX15 | MIM | OXPHOS > Complex IV > CIV assembly factors \| Metabolism > Metals and cofactors > Heme synthesis and processing \| OXPHOS > OXPHOS assembly factors |
| SURF1 | MIM | OXPHOS > Complex IV > CIV assembly factors \| OXPHOS > OXPHOS assembly factors |
| COA5 | IMS | OXPHOS > Complex IV > CIV assembly factors \| OXPHOS > OXPHOS assembly factors |
| COX6C | MIM | OXPHOS > Complex IV > CIV subunits \| OXPHOS > OXPHOS subunits |
| TACO1 | Matrix | Mitochondrial central dogma > Translation > Translation factors \| OXPHOS > Complex IV > CIV assembly factors \| OXPHOS > OXPHOS assembly factors |
| CMC1 | IMS | OXPHOS > Complex IV > CIV assembly factors \| OXPHOS > OXPHOS assembly factors |
| ATP5PD | MIM | OXPHOS > Complex V > CV subunits \| OXPHOS > OXPHOS subunits |
| HADH | Matrix | Metabolism > Lipid metabolism > Fatty acid oxidation \| Metabolism > Amino acid metabolism > Lysine metabolism |
| MECR | Matrix | Metabolism > Lipid metabolism > Type II fatty acid synthesis |
| FDX1 | Matrix | Metabolism > Lipid metabolism > Cholesterol, bile acid, steroid synthesis \| Metabolism > Metals and cofactors > Heme synthesis and processing \| Metabolism > Metals and cofactors > Fe-S cluster biosynthesis \| Metabolism > Metals and cofactors > Fe-S-containing proteins \| Metabolism > Vitamin metabolism > Vitamin D metabolism |
| CRAT | MIM | Metabolism > Lipid metabolism > Fatty acid oxidation |
| FDXR | Matrix | Metabolism > Lipid metabolism > Cholesterol, bile acid, steroid synthesis \| Metabolism > Metals and cofactors > Heme synthesis and processing \| Metabolism > Metals and cofactors > Fe-S cluster biosynthesis \| Metabolism > Vitamin metabolism > Vitamin D metabolism |
| CBR4 | Matrix | Metabolism > Lipid metabolism > Type II fatty acid synthesis |
| HINT2 | Matrix | Metabolism > Lipid metabolism > Cholesterol, bile acid, steroid synthesis |
| DECR1 | Matrix | Metabolism > Lipid metabolism > Fatty acid oxidation |
| MCEE | Matrix | Metabolism > Carbohydrate metabolism > Propanoate metabolism \| Metabolism > Lipid metabolism > Fatty acid oxidation |
| ACSM1 | Matrix | Metabolism > Lipid metabolism > Fatty acid oxidation |
| EHHADH | unknown | Metabolism > Lipid metabolism |
| HSD17B10 | Matrix | Metabolism > Lipid metabolism > Fatty acid oxidation \| Metabolism > Lipid metabolism > Cholesterol, bile acid, steroid synthesis \| Metabolism > Amino acid metabolism > Branched-chain amino acid metabolism |
| ECI1 | Matrix | Metabolism > Lipid metabolism > Fatty acid oxidation |
| HSD17B4 | unknown | Metabolism > Lipid metabolism |
| CHPT1 | MIM | Metabolism > Lipid metabolism > Phospholipid metabolism |
| PRDX6 | unknown | Metabolism > Lipid metabolism > Phospholipid metabolism \| Metabolism > Detoxification > ROS and glutathione metabolism |
| LYPLAL1 | unknown | Metabolism > Lipid metabolism |
| ACACB | MOM | Metabolism > Lipid metabolism > Fatty acid oxidation \| Metabolism > Vitamin metabolism > Biotin utilizing proteins |
| ACSL1 | MOM | Metabolism > Lipid metabolism > Fatty acid oxidation |
| SCP2 | MIM | Metabolism > Lipid metabolism > Cholesterol, bile acid, steroid synthesis |
| CYP11A1 | MIM | Metabolism > Lipid metabolism > Cholesterol, bile acid, steroid synthesis \| Metabolism > Metals and cofactors > Heme-containing proteins |
| THEM5 | Matrix | Metabolism > Lipid metabolism |
| ACSM2B | Matrix | Metabolism > Lipid metabolism > Fatty acid oxidation |
| GPAT2 | MOM | Metabolism > Lipid metabolism > Cardiolipin synthesis \| Metabolism > Lipid metabolism > Phospholipid metabolism |

Mitochondrion compartments: matrix, MIM (mitochondrial inner membrane), IMS (intermembrane space), MOM (mitochondrial outer membrane). Green highlighting refers to mitochondrion-associated genes downregulated in high miR-494 expressing HCCs. Yellow highlighting refers to lipid metabolism-associated genes downregulated in high miR-494 expressing HCCs.

# Supplementary Table 9 – Metabolic gene deregulation in human HCC cohorts

| GENE SYMBOL | TCGA-HCC | | Bologna HCC cohort | |
| --- | --- | --- | --- | --- |
|  | **LogFC** | **p-value** | **LogFC** | **p-value** |
| G6PD | 2,35 | 7,27E-26 | 1,26 | 2,51E-02 |
| FASN | 1,13 | 1,22E-06 | 0,53 | 2,63E-01 |
| LPIN1 | 0,42 | 1,20E-02 | 0,50 | 3,96E-02 |
| ETFDH | -1,39 | 6,13E-22 | -1,25 | 4,42E-04 |
