# Supplementary material for: MiR-494 induces metabolic changes through G6pc targeting and modulates sorafenib response in hepatocellular carcinoma
Source: J Exp Clin Cancer Res. 2023 Jun 10;42:145. doi: 10.1186/s13046-023-02718-w (PMC10257313; doi:10.1186/s13046-023-02718-w)
Supplement: Supplementary file 2 — Additional file 2. Supplementary Figures. [file 13046_2023_2718_MOESM2_ESM.docx]

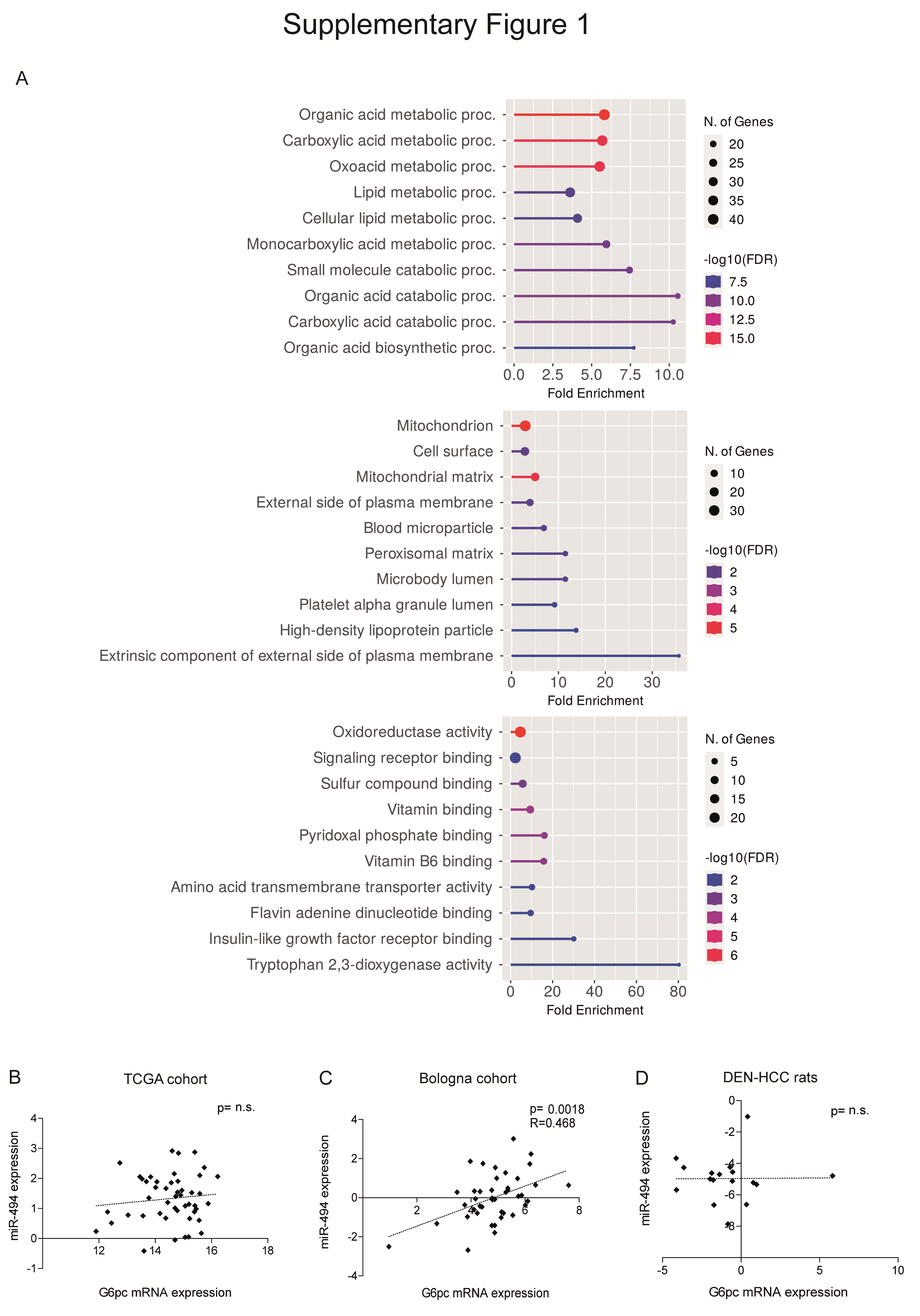


**Figure S1.** (**A**) Gene ontology (GO) enrichment analysis of the 144 common genes from Figure 1A. The lollypop plots (from top to bottom) show the top 10 most enriched GO Biological Process (BP), GO Cellular Component (CC) and GO Molecular Function (MF). (**B-D**) Correlation graphs between miR-494 and G6pc mRNA levels in surrounding liver tissues of the TCGA (N=47) and Bologna (N=42) cohorts and DEN-HCC rats (N=17). Axes report 2^-ΔΔCt^ values corresponding to miR-494 and G6pc expression levels transformed in a log2 form. N.s. = not significant. U6RNA, GAPDH and Beta-actin were used as housekeeping genes for miRNA and mRNA analysis, respectively. Real Time PCR was run in triplicate.


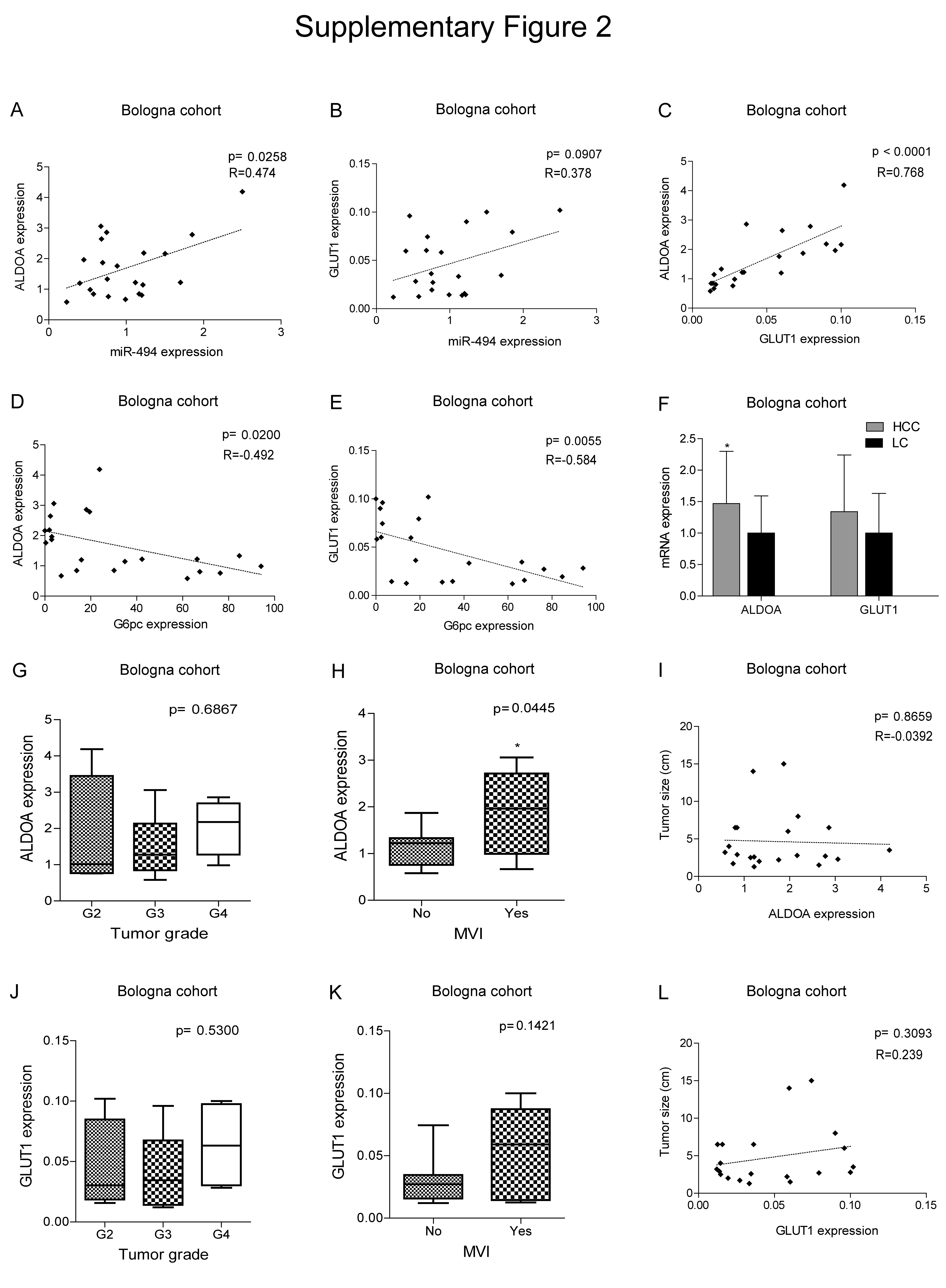


**Figure S2**. **Correlation** **of HIF-1A transcriptional targets with miR-494 and G6pc expression and association with clinicopathological features in human HCCs**. (**A**, **B**) Correlation graphs between miR-494 expression and ALDOA or GLUT1 mRNA levels in HCC tissues (N=22) from the Bologna patient cohort. (**C**) Correlation graphs between ALDOA and GLUT1 mRNA levels in HCC tissues (N=22) from the Bologna cohort. (**D**, **E**) Correlation graphs between G6pc and ALDOA or GLUT1 mRNA levels in HCC tissues (N=22) from the Bologna patient cohort. (**A-E**) Axes report 2^-ΔΔCt^ values corresponding to miR-494 expression and mRNA levels of G6pc and HIF-1A target genes. U6RNA and GAPDH were used as housekeeping genes for miRNA and mRNA expression, respectively. Real Time PCR was run in triplicate. (**F**) Histogram graph representing ALDOA and GLUT1 mRNA levels in matched HCC and liver cirrhosis (LC) tissues (N=22) from the Bologna patient cohort. Y-axis reports 2^-ΔΔCt^ values corresponding to mRNA levels of HIF-1A target genes normalized to LC. Mean ± SD values are reported. GAPDH was used as housekeeping gene. Real Time PCR was run in triplicate. (**G**) Box plot graph of ALDOA mRNA levels in HCC specimens (N=22) from the Bologna patient cohort divided according to tumor grade. On the top of the graph is reported the p-value relative to ANOVA. (**H**) Box plot graph of ALDOA mRNA levels in HCC specimens (N=22) from the Bologna patient cohort divided according to the presence or absence of microvascular invasion (MVI). Y-axes report 2^-ΔΔCt^ values corresponding to mRNA levels. GAPDH was used as housekeeping gene. Real Time PCR was run in triplicate. (**I**) Correlation graph between ALDOA mRNA levels and tumor size of HCC nodules (N=22) from the Bologna patient cohort. Y-axes report 2^-ΔΔCt^ values corresponding to mRNA levels and tumor nodule diameter (cm). GAPDH was used as housekeeping gene. Real Time PCR was run in triplicate. (**J**) Box plot graph of GLUT1 mRNA levels in HCC specimens (N=22) from the Bologna patient cohort divided according to tumor grade. On the top of the graph is reported the p-value relative to ANOVA. (**K**) Box plot graph of GLUT1 mRNA levels in HCC specimens (N=22) from the Bologna cohort divided according to the presence or absence of microvascular invasion (MVI). Y-axes report 2^-ΔΔCt^ values corresponding to mRNA levels. GAPDH was used as housekeeping gene. Real Time PCR was run in triplicate. (**L**) Correlation graph between GLUT1 mRNA levels and tumor size of HCC nodules (N=22) from the Bologna patient cohort. Y-axes report 2^-ΔΔCt^ values corresponding to mRNA levels and tumor nodule diameter (cm). GAPDH was used as housekeeping gene. Real Time PCR was run in triplicate.

Two-tailed unpaired Student's t-test and Pearson’s correlation were used. * *P* ≤ 0.05.


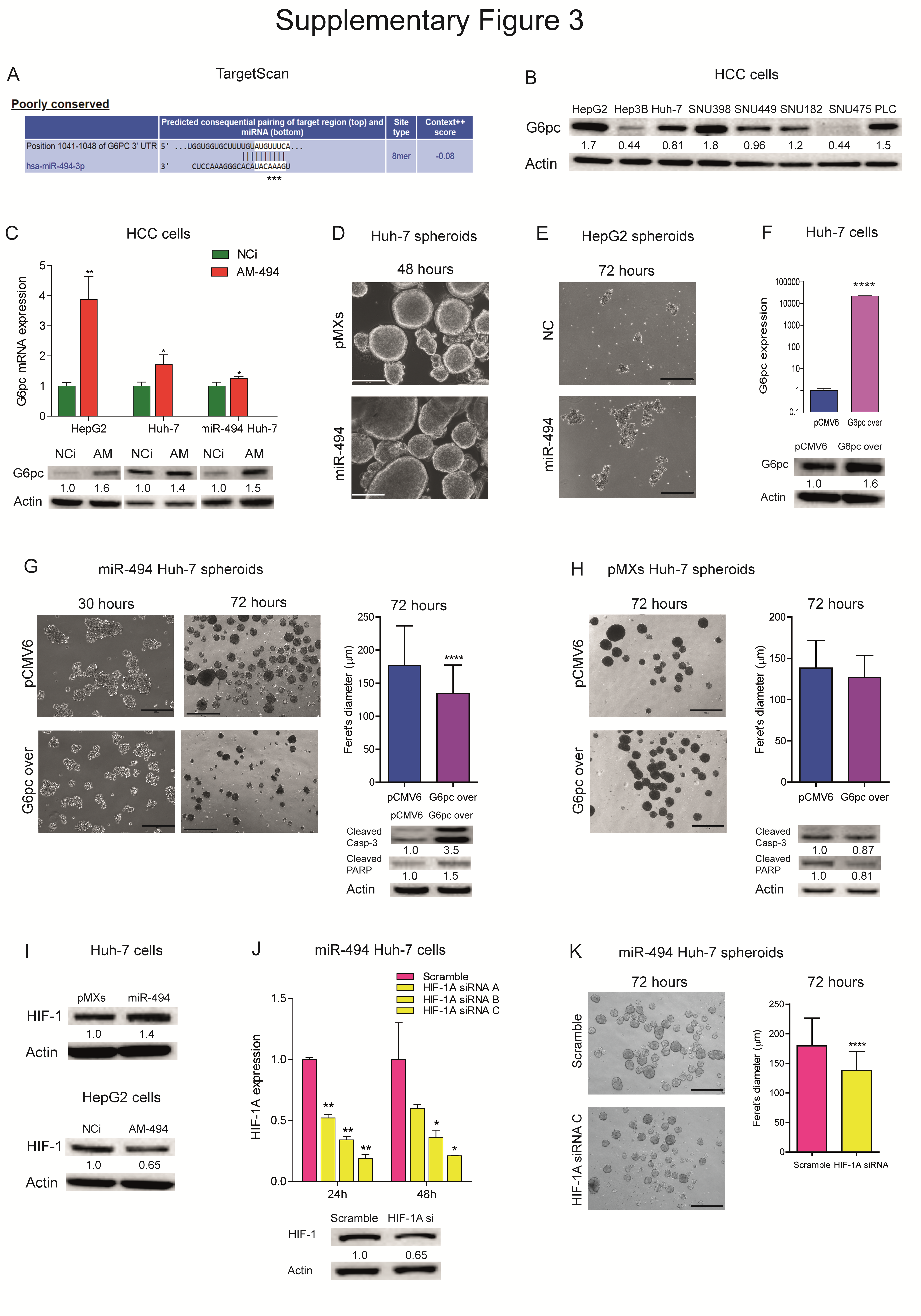


**Figure S3.** (**A**) Complementary miR-494 binding site in *G6PC* 3’UTR, as displayed by TargetScan. Stars below the seed sequence represent mutated bases in the mutated vector used in the dual-luciferase reporter assay. (**B**) WB analysis of G6pc protein levels in ~~eight~~ HCC-derived cell lines (N=8). Beta-actin was used as housekeeping gene. (**C**) Real Time PCR and WB analyses of G6pc expression following miR-494 inhibition in transiently transfected HCC cells and miR-494-overexpressing Huh-7 cells. NCi: negative control inhibitor miRNA; AM-494: antimiR-494. Y-axis reports 2^-ΔΔCt^ values corresponding to G6pc mRNA levels normalized to controls. Mean ± SD values are displayed. Beta-actin was used as housekeeping gene for Real Time and WB experiments. Real Time PCR analysis was performed ~~twice~~ in two independent experiments in triplicate; WB analysis was performed in two independent experiments. (**D**) Representative images (20X magnification) of miR-494-overexpressing (miR-494) and control (pMXs) Huh-7 spheroids at 48 hours. Two independent experiments were performed. Scale bars, 150 μm. (**E**) Representative images (4X magnification) of miR-494 and control (NC) HepG2 spheroids at 72 hours. Two independent experiments were performed. Scale bars, 750 μm. (**F**) Real Time PCR and WB analyses of G6pc expression in Huh-7 cells following transfection with G6pc overexpressing (G6pc over) or control (pCMV6) vector. Y-axis reports 2^-ΔΔCt^ values corresponding to G6pc mRNA levels normalized to control. Mean ± SD values are displayed. Real Time PCR analysis was performed in two independent experiments in triplicate. WB was performed in two independent experiments. (**G**) On the left: representative images (10X magnification) of miR-494-overexpressing Huh-7 spheroids obtained following transfection with G6pc overexpression (G6pc over) or control (pCMV6) vector at 30 hours. Scale bars, 300 μm. On the the right: representative images (4X magnification) of miR-494-overexpressing Huh-7 spheroids obtained following transfection with G6pc overexpression (G6pc over) or control (pCMV6) vector at 72 hours. Data were obtained by measuring feret’s diameter (µm) of thirty randomly selected spheroids from two independent experiments. Mean ± SD values are displayed. Scale bars, 750 μm. WB analysis of cleaved caspase-3 and PARP in miR-494-overexpressing Huh-7 spheroids obtained after transfection with G6pc overexpression (G6pc over) or control (pCMV6) vector (72 hours). Beta-actin was used as housekeeping gene. Two independent experiments were performed. (**H**) Representative images (4X magnification) of control (pMXs) Huh-7 spheroids obtained following transfection with G6pc overexpression (G6pc over) or control (pCMV6) vector at 72 hours. Data were obtained by measuring feret’s diameter (µm) of thirty randomly selected spheroids from two independent experiments. Mean ± SD values are displayed. Scale bars, 750 μm. WB analysis of cleaved caspase-3 and PARP in control (pMXs) Huh-7 spheroids obtained after transfection with G6pc overexpression (G6pc over) or control (pCMV6) vector. Two independent experiments were performed. (**I**) WB analysis of HIF-1A protein levels in miR-494-overexpressing Huh-7 cells and in antimiR-494 (AM-494) transfected HepG2 cells. Beta-actin was used as housekeeping gene. PMXs: control vector; NCi: negative control inhibitor miRNA. Two independent experiments were performed. (**J**) Real Time PCR and WB analyses of HIF-1A in miR-494-overexpressing Huh-7 cells following transfection with HIF-1A DsiRNAs and scramble oligonucleotides. Y-axis reports 2^-ΔΔCt^ values corresponding to HIF-1A mRNA levels normalized to control. Mean ± SD values are displayed. Real Time PCR analysis was performed in two independent experiments in triplicate. WB was performed at 48 hours with HIF-1A DsiRNA C (HIF-1A si). Two independent experiments were performed. (**K**) Representative images (4X magnification) of miR-494-overexpressing Huh-7 spheroids obtained following transfection with HIF-1A DsiRNA C (HIF-1A siRNA) or scramble oligonucleotides at 72 hours. Data were obtained by measuring feret’s diameter (µm) of thirty randomly selected spheroids from two independent experiments. Mean ± SD values are displayed. Scale bars, 750 μm. Two-tailed Student's t-test and Pearson’s correlation were used. * *P* ≤ 0.05; *** P* ≤ 0.01; **** *P* ≤ 0.0001.


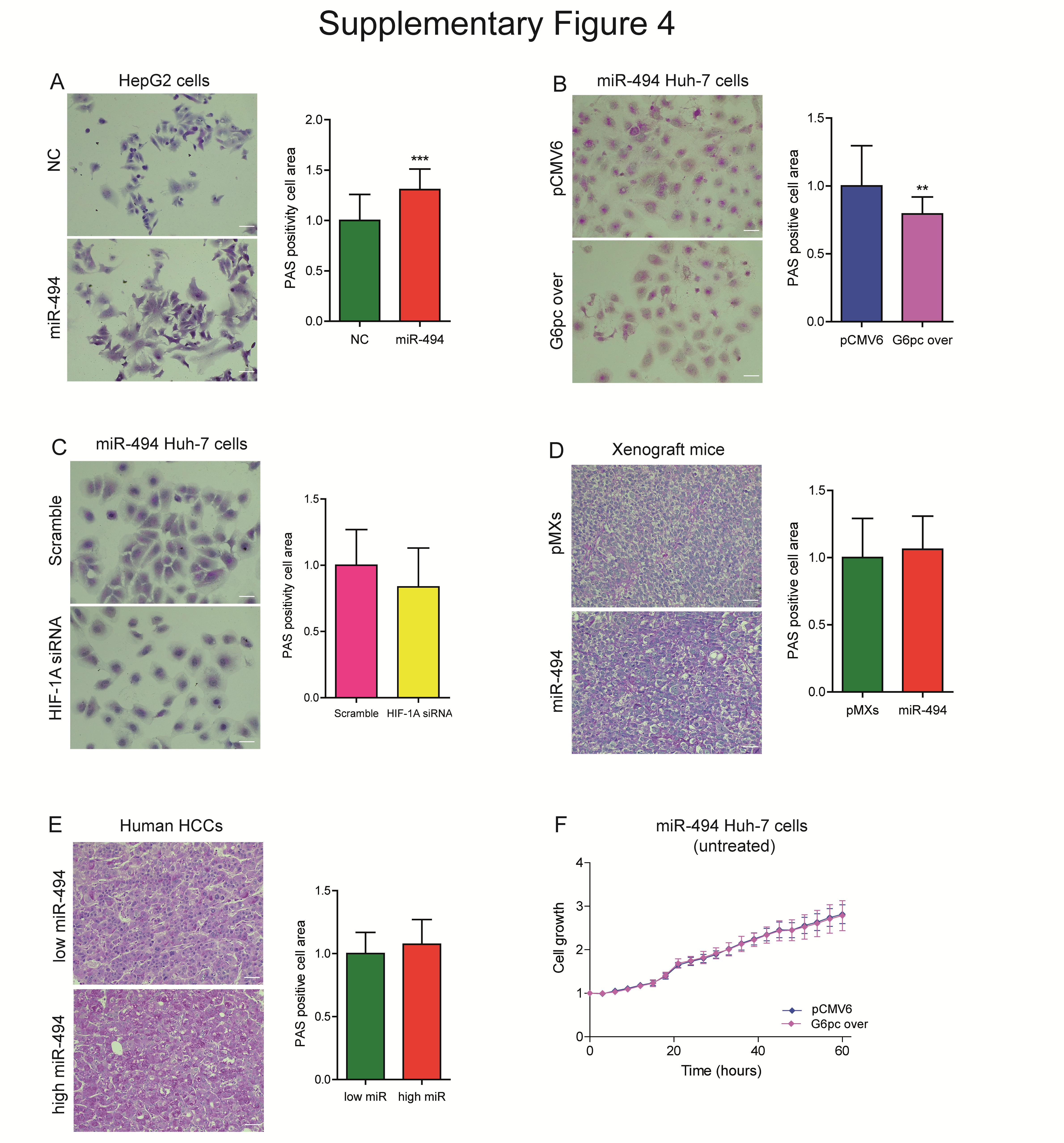


**Figure S4.** (**A**) Representative images (20X magnification) of PAS staining after miR-494 or negative control (NC) transfection in HepG2 cells. Y-axis reports the percent of PAS positive cell area normalized to control. Mean ± SD values are displayed. Five randomly selected fields were analyzed. Three independent experiments were performed. Scale bars, 50 μm. (**B**) Representative images (20X magnification) of PAS staining in miR-494-overexpressing Huh-7 cells following transfection with control (pCMV6) and G6pc overexpressing (G6pc over) vectors. Y-axis reports the percent of PAS positive cell area normalized to control. Mean ± SD values are displayed. Five randomly selected fields were analyzed. Three independent experiments were performed. Scale bars, 50 μm. (**C**) Representative images (20X magnification) of PAS staining in miR-494-overexpressing Huh-7 cells following transfection with control (scramble) or HIF-1A DsiRNA C (HIF-1A siRNA) oligonucleotides. Y-axis reports the percent of PAS positive cell area normalized to control. Mean ± SD values are displayed. Five randomly selected fields were analyzed. Three independent experiments were performed. Scale bars, 50 μm. (**D**) Representative images (20X magnification) of PAS staining in xenograft tumors (N=11) derived from control (pMXs) and miR-494-overexpressing Huh-7 cells. Y-axis reports the percent of PAS positive cell area normalized to controls. Mean ± SD values are displayed. Five randomly selected fields were analyzed for each sample. Scale bars, 50 μm. (**E**) Representative images (20X magnification) of PAS staining in high and low miR-494 expressing HCC tissues (N=10). Y-axis reports the percent of PAS positive cell area normalized to low miRNA-expressing samples. Mean ± SD values are displayed. Five randomly selected fields were analyzed for each sample. Scale bars, 50 μm. (**F**) Growth curves of miR-494-overexpressing Huh-7 cells transfected with G6pc overexpression (G6pc over) or control (pCMV6) vector and grown in standard culture conditions. Growth curves were normalized to T0. Mean ± SD values are reported. Two independent experiments were performed in quadruplicate.

Statistical significance was determined by two-tailed unpaired Student's t-test. ** *P* ≤ 0.01; *** *P* ≤ 0.001.


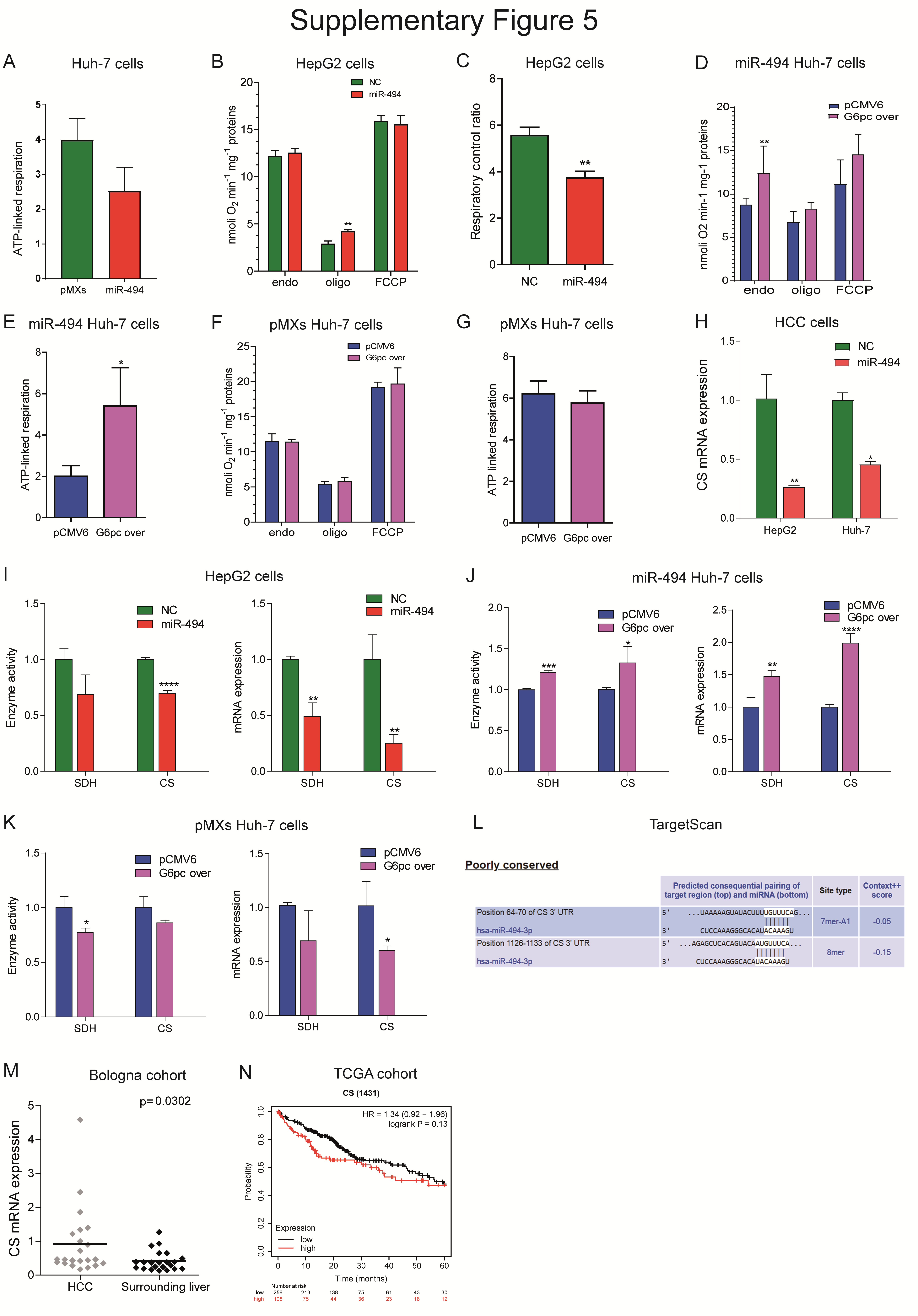


**Figure S5.** (**A**) ATP-linked respiration derived from the difference between basal oxygen consumption rate and oxygen consumption rate following oligomycin addition in control (pMXs) and miR-494-overexpressing Huh-7 cells. Mean ± SD values are displayed. Three independent experiments were performed. (**B**) Oxygen consumption rate after miR-494 or negative control (NC) transfection in HepG2 cells measured in standard medium (endogenous respiration); in the presence of oligomycin A (Oligo) and carbonyl cyanide 4-(trifluoromethoxy) phenylhydrazone (FCCP). Mean ± SD values are displayed. Three independent experiments were performed. (**C**) ATP-linked respiration in miR-494 or negative control (NC) transfected HepG2 cells. Mean ± SD values are displayed. Three independent experiments were performed. (**D**) Oxygen consumption rate in miR-494-overexpressing Huh-7 cells following transfection with G6pc overexpression (G6pc over) and control (pCMV6) vectors measured in standard medium (endogenous respiration); in the presence of oligomycin A (Oligo) and carbonyl cyanide 4-(trifluoromethoxy) phenylhydrazone (FCCP). Mean ± SD values are displayed. Three independent experiments were performed. (**E**) ATP-linked respiration in miR-494-overexpressing Huh-7 cells following transfection with G6pc overexpression (G6pc over) and control (pCMV6) vectors. Mean ± SD values are displayed. Three independent experiments were performed. (**F**) Oxygen consumption rate in control (pMXs) Huh-7 cells following transfection with G6pc overexpression (G6pc over) and control (pCMV6) vectors measured in standard medium (endogenous respiration); in the presence of oligomycin A (Oligo) and carbonyl cyanide 4-(trifluoromethoxy) phenylhydrazone (FCCP). Mean ± SD values are displayed. Three independent experiments were performed. (**G**) ATP-linked respiration in control (pMXs) Huh-7 cells following transfection with G6pc overexpression (G6pc over) and control (pCMV6) vectors. Mean ± SD values are displayed. Three independent experiments were performed. (**H**) Real Time PCR analysis of citrate synthase (CS) expression following miR-494 transfection in HCC cells. NC: negative control precursor miRNA. Y-axis reports 2^-ΔΔCt^ values corresponding to mRNA levels normalized to controls. Beta-actin was used as housekeeping gene. Mean ± SD values are displayed. Real Time PCR analysis was performed in two independent experiments in triplicate. (**I**) Enzymatic activity and real time PCR analysis of succinate dehydrogenase (SDH) and citrate synthase (CS) after miR-494 or negative control (NC) transfection in HepG2 cells. The Y-axes report the enzymatic activity (µmol*min^-1^*mg^-1^) and 2^-ΔΔCt^ values normalized to control. Mean ± SD values are displayed. Three independent experiments were performed. Real Time PCR analysis was performed in two independent experiments in triplicate. (**J**) Enzymatic activity and real time PCR analysis of succinate dehydrogenase (SDH) and citrate synthase (CS) in miR-494-overexpressing or (**K**) control (pMXs) Huh-7 cells following transfection with control (pCMV6) and G6pc overexpressing (G6pc over) vectors. The Y-axes report the enzymatic activity (µmol*min^-1^*mg^-1^) and 2^-ΔΔCt^ values normalized to control. Mean ± SD values are displayed. Three independent experiments were analyzed for the enzyme activity assay. Real Time PCR analysis was performed in two independent experiments in triplicate. (**L**) Complementary miR-494 binding sites in *CS* 3’UTR, as displayed by TargetScan algorithm. (**M**) Box plot graph of CS mRNA levels in HCC and surrounding livers of patients (N=22) from the Bologna cohort. Y-axis reports 2^-ΔΔCt^ values corresponding to mRNA levels. Beta-actin was used as housekeeping gene. Real Time PCR analysis was run in triplicate. (**N**) Kaplan-Meier curves of high and low CS-expressing HCCs (TCGA cohort).

Statistical significance was determined by two-tailed unpaired Student's t-test. * *P* ≤ 0.05; ** *P* ≤ 0.01; **** *P* ≤ 0.0001.


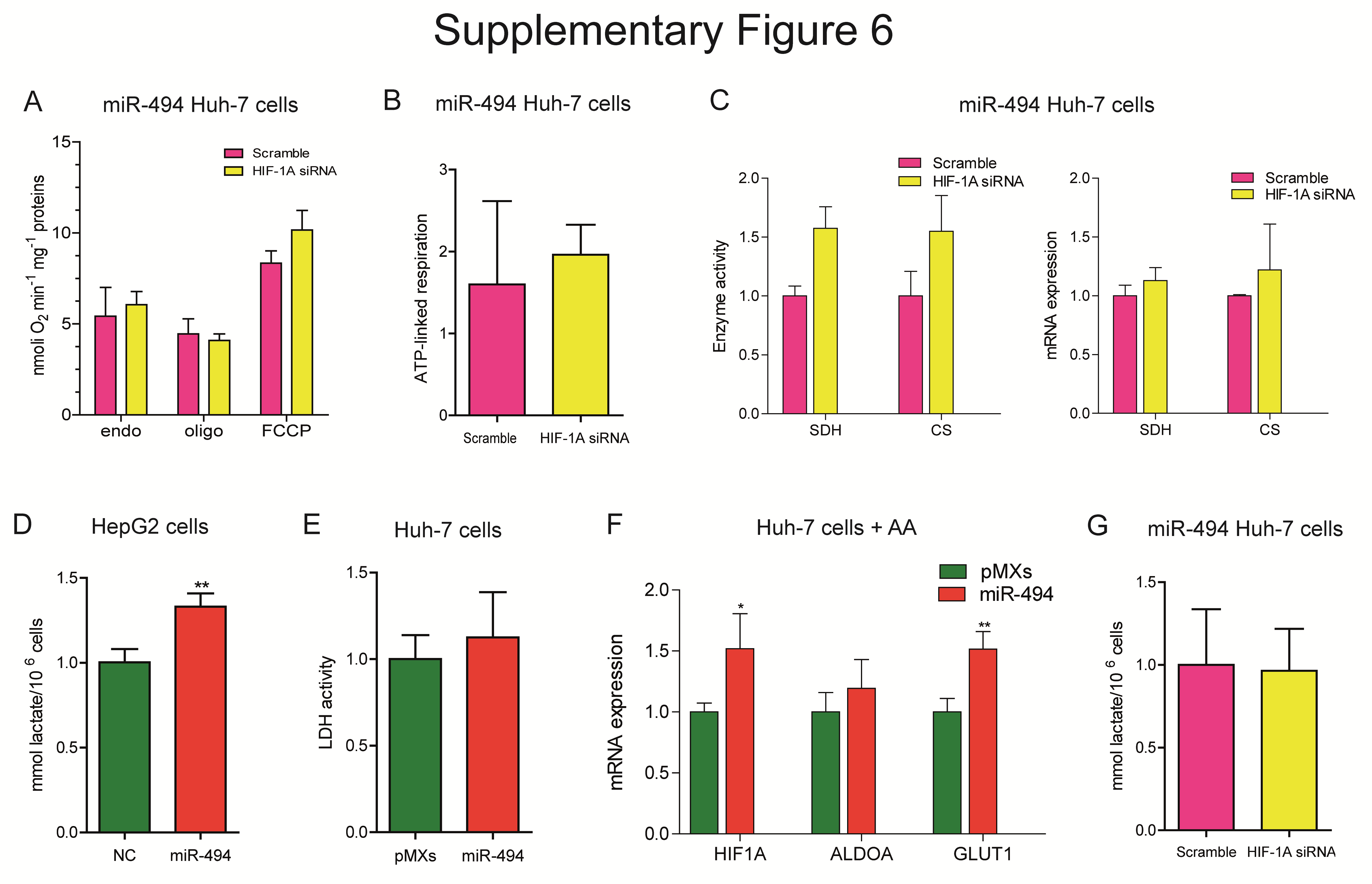


**Figure S6.** (**A**) Oxygen consumption rate in miR-494-overexpressing Huh-7 cells following transfection with HIF-1A DsiRNAs C (HIF-1A siRNA) and scramble oligonucleotides measured in standard medium (endogenous respiration); in the presence of oligomycin A (Oligo) and carbonyl cyanide 4-(trifluoromethoxy) phenylhydrazone (FCCP). Mean ± SD values are displayed. Three independent experiments were performed. (**B**) ATP-linked respiration in miR-494-overexpressing Huh-7 cells following transfection with HIF-1A DsiRNAs C (HIF-1A siRNA) and scramble oligonucleotides. Mean ± SD values are displayed. Three independent experiments were performed. (**C**) Enzymatic activity and real time PCR analysis of succinate dehydrogenase (SDH) and citrate synthase (CS) in miR-494-overexpressing Huh-7 cells following transfection with HIF-1A DsiRNAs C (HIF-1A siRNA) and scramble oligonucleotides. The Y-axes report the enzymatic activity (µmol*min^-1^*mg^-1^) and 2^-ΔΔCt^ values normalized to control. Mean ± SD values are displayed. Three independent experiments were performed. Real Time PCR analysis was performed in two independent experiments in triplicate. (**D**) Extracellular lactate quantification (mmol lactate/10^6^ cells) after negative control (NC) or miR-494 transfection in HepG2 cells. Normalized mean ± SD values are displayed. Three independent experiments were performed. (**E**) Enzymatic activity (nmol*min^-1^*mg^-1^) of lactate dehydrogenase (LDH) in control (pMXs) and miR-494-overexpressing Huh-7 cells. Normalized mean ± SD values are displayed. Three independent experiments were analyzed in duplicate. (**F**) Real Time PCR analysis of HIF1A gene and its metabolic targets in control (pMXs) and miR-494-overexpressing Huh-7 cells in the presence of antimycin A (AA). Y-axis reports 2^-ΔΔCt^ values corresponding to mRNA levels normalized to controls. Beta-actin was used as housekeeping gene. Mean ± SD values are displayed. Real Time PCR analysis was ~~run~~ performed in two independent experiments in triplicate. (**G**) Extracellular lactate quantification (mmol lactate/10^6^ cells) in miR-494-overexpressing Huh-7 cells following transfection with HIF-1A DsiRNAs C (HIF-1A siRNA) and scramble oligonucleotides. Normalized mean ± SD values are displayed. Three independent experiments were performed.

Statistical significance was determined by two-tailed unpaired Student's t-test. * *P* ≤ 0.05; ** *P* ≤ 0.01.


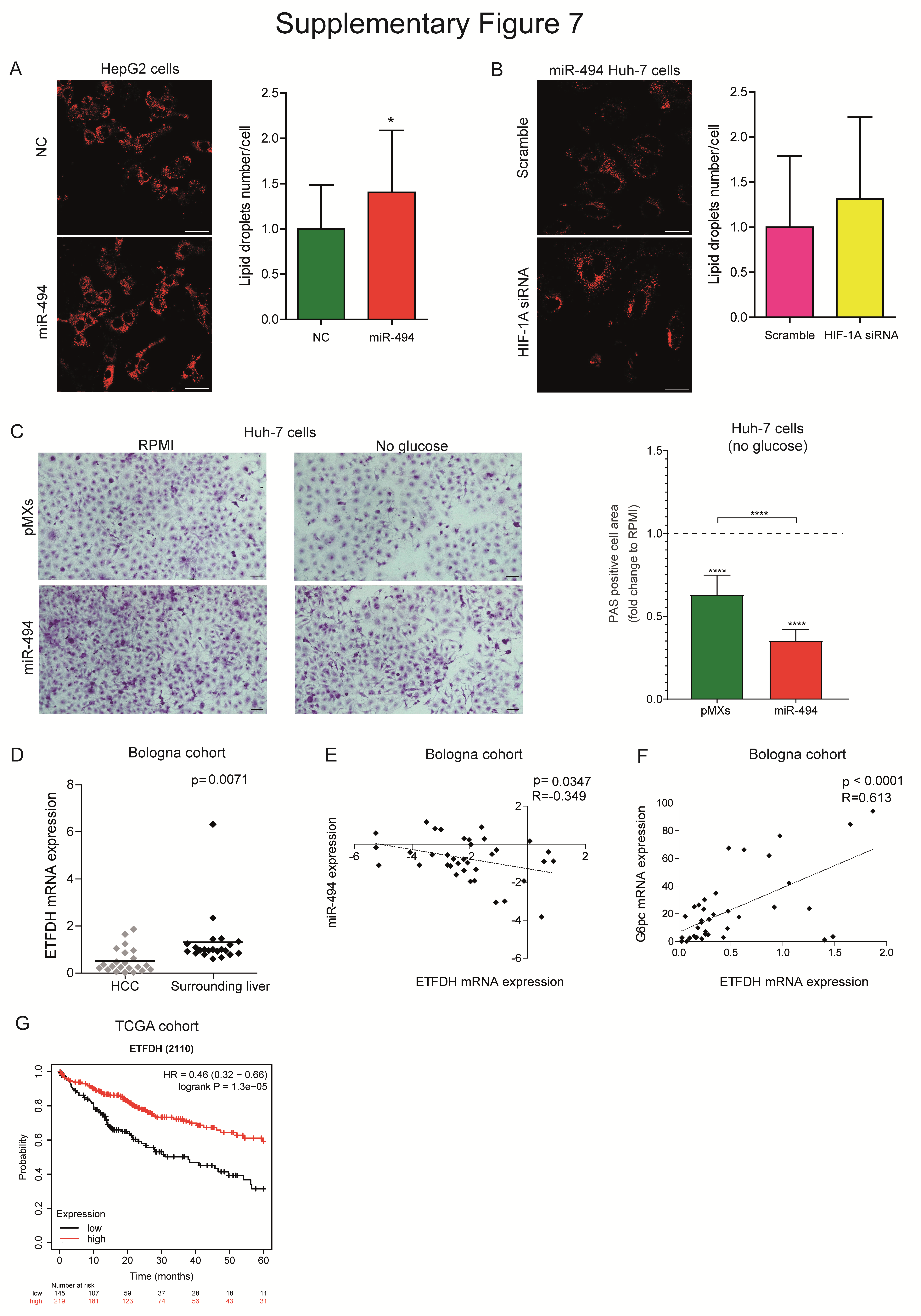


**Figure S7.** (**A**) Representative confocal images of lipid droplets (LDs) after negative control (NC) or miR-494 transfection in HepG2 cells stained with Nile Red. The Y-axis shows the quantification of LDs number per cell normalized to control. Mean ± SD values are reported. Two independent experiments were performed. Scale bar = 20 µm. (**B**) Representative confocal images of lipid droplets (LDs) in miR-494-overexpressing Huh-7 cells after transfection with HIF-1A DsiRNA C (HIF-1A siRNA) or control (scramble) oligonucleotides stained with Nile Red. The Y-axis shows the quantification of LDs number per cell normalized to control. Mean ± SD values are reported. Two independent experiments were performed. Scale bar, 20 µm. (**C**) Representative images (20X magnification) of PAS staining in control (pMXs) and miR-494 Huh-7 cells grown in standard medium (RPMI) or in no glucose medium. Data are displayed as the fold change of PAS staining (% of PAS positive cell area) in Huh-7 cells grown in glucose-free medium with respect to RPMI. Five randomly selected fields were analyzed from three independent experiments. Scale bars, 20 μm. (**D**) Box plot graph of ETFDH mRNA levels in HCC and surrounding livers of patients (N=22) from the Bologna cohort. Y-axis reports 2^-ΔΔCt^ values corresponding to mRNA levels. GAPDH was used as housekeeping gene. Real Time PCR analysis was run in triplicate. (**E**) Correlation graph between ETFDH and G6pc mRNA levels or (**F**) between miR-494 and ETFDH in HCC tissues of patients (N=36) from the Bologna cohort. Axes report 2^-ΔΔCt^ values corresponding to miR-494, G6pc and ETFDH expression levels. Values in miR-494/ETFDH correlation graph are transformed in a log2 form. U6RNA and GAPDH were used as housekeeping genes. Real Time PCR analysis was run in triplicate. (**G**) Kaplan-Meier curves of high and low ETFDH-expressing HCCs (TCGA cohort; N=364).

Statistical significance was determined by two-tailed unpaired Student's t-test and Pearson’s correlation. * *P* ≤ 0.05; **** *P* ≤ 0.0001.


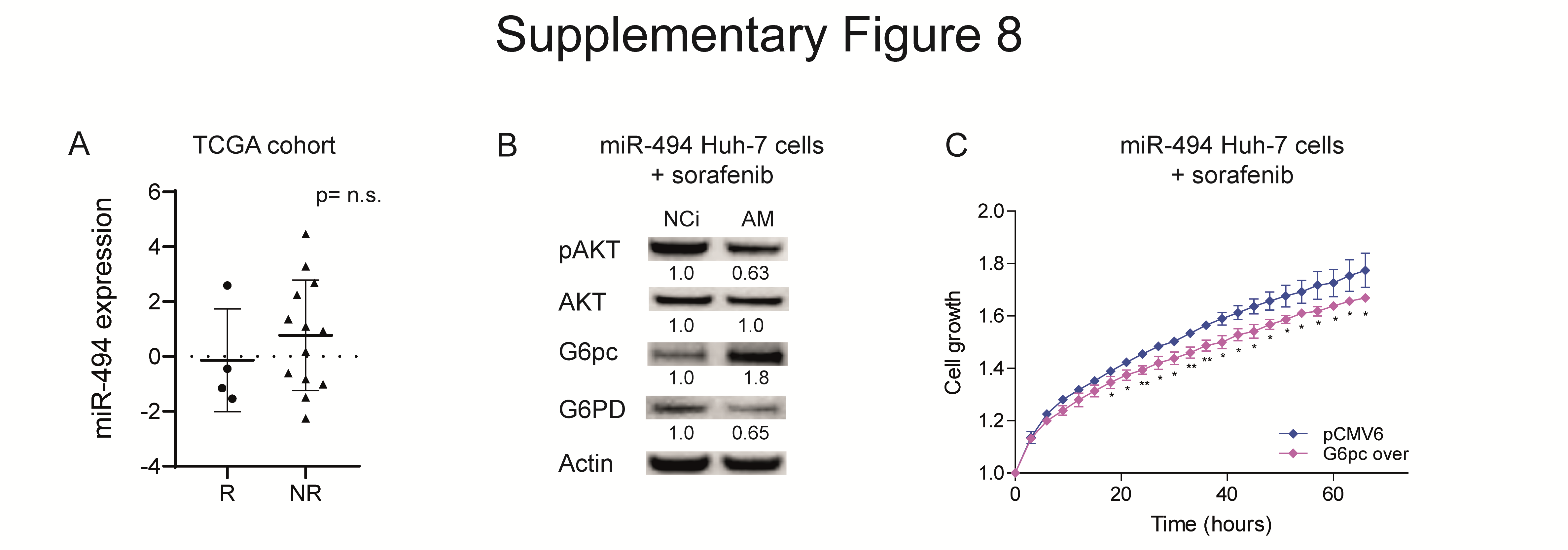


**Figure S8.** (**A**) Box plot graph of miR-494 expression in HCC tissues from responder (R) and non-responder (NR) sorafenib-treated patients (N=17) of the TCGA-HCC cohort. N.s. = not significant. (**B**) WB analysis of phosphorylated and total AKT levels, G6pc and G6PD levels in miR-494-overexpressing Huh-7 cells transfected with antimiR-494 (AM-494) or negative control (NCi) and subjected to sorafenib treatment for 48 hours. Beta-actin was used as housekeeping gene. Two independent experiments were performed. (**C**) Growth curves of miR-494-overexpressing Huh-7 cells transfected with G6pc overexpressing (G6pc over) or control (pCMV6) vector and cultured in the presence of sorafenib (5 µM). Growth curves were normalized to T0. Mean ± SD values are reported. Two independent experiments were performed in quadruplicate.

The statistical analysis was performed using two-tailed unpaired Student's t-test. * *P* ≤ 0.05; ** *P* ≤ 0.01.
